# Molecular Architecture of the 40S⋅eIF1⋅eIF3 Translation Initiation Complex

**DOI:** 10.1016/j.cell.2014.07.044

**Published:** 2014-08-28

**Authors:** Jan P. Erzberger, Florian Stengel, Riccardo Pellarin, Suyang Zhang, Tanja Schaefer, Christopher H.S. Aylett, Peter Cimermančič, Daniel Boehringer, Andrej Sali, Ruedi Aebersold, Nenad Ban

**Affiliations:** 1 Department of Biology, Institute of Molecular Biology and Biophysics, ETH Zurich, Otto-Stern-Weg 5, 8093 Zurich, Switzerland; 2 Department of Biology, Institute of Molecular Systems Biology, ETH Zurich, Auguste-Piccard-Hof 1, 8093 Zurich, Switzerland; 3 Department of Bioengineering and Therapeutic Sciences, Department of Pharmaceutical Chemistry, and California Institute for Quantitative Biosciences (QB3), University of California, San Francisco, UCSF MC 2552, Byers Hall Room 503B, 1700 4th Street, San Francisco, CA 94158-2330, USA; 4 Faculty of Science, University of Zurich, 8006 Zurich, Switzerland

## Abstract

Eukaryotic translation initiation requires the recruitment of the large, multiprotein eIF3 complex to the 40S ribosomal subunit. We present X-ray structures of all major components of the minimal, six-subunit *Saccharomyces cerevisiae* eIF3 core. These structures, together with electron microscopy reconstructions, cross-linking coupled to mass spectrometry, and integrative structure modeling, allowed us to position and orient all eIF3 components on the 40S⋅eIF1 complex, revealing an extended, modular arrangement of eIF3 subunits. Yeast eIF3 engages 40S in a clamp-like manner, fully encircling 40S to position key initiation factors on opposite ends of the mRNA channel, providing a platform for the recruitment, assembly, and regulation of the translation initiation machinery. The structures of eIF3 components reported here also have implications for understanding the architecture of the mammalian 43S preinitiation complex and the complex of eIF3, 40S, and the hepatitis C internal ribosomal entry site RNA.

## Highlights

• X-ray structures of major yeast eIF3 components and subcomplexes
• Crosslinking coupled to mass-spectrometry analysis of 40S⋅eIF1⋅eIF3 complex
• Integrative modeling reveals architecture of 40S⋅eIF1⋅eIF3 complex

## Graphical Abstract

**Figure:**
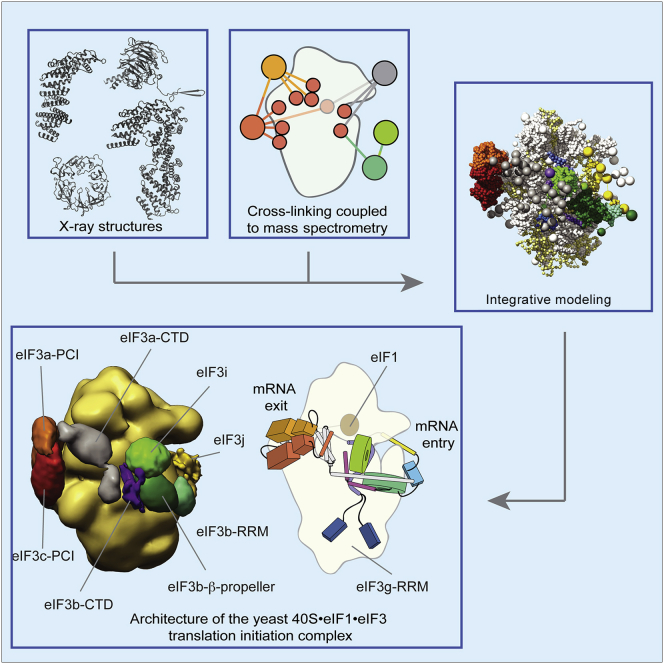

## Teaser

A hybrid approach drawing on X-ray structures, crosslinking coupled to mass spectrometry, electron microscopy, and integrative modeling yields mechanistic insights into how eIF3 coordinates translation initiation.

## Introduction

Protein synthesis is catalyzed by the ribosome in a process that consists of initiation, elongation, and termination. In bacteria, three initiation factors (IF1, IF2, IF3) and the Shine-Dalgarno sequence are sufficient to accurately pair the AUG start codon in messenger RNA (mRNA) with the anticodon loop of methionyl initiator transfer RNA (Met-tRNAi). Eukaryotes, in contrast, require at least 25 different polypeptides assembled into eight eukaryotic initiation factors (eIFs) to initiate protein synthesis and employ a complex scanning mechanism to probe the 5′ leader sequences of mRNA for the correct start site (Hinnebusch, 2011, Hinnebusch, 2014, Jackson et al., 2010, Voigts-Hoffmann et al., 2012). Initiation is targeted by a number of regulatory pathways linked to cellular processes such as cell growth, differentiation, and environmental stress responses (Sonenberg and Hinnebusch, 2009), and the functional disruption or decoupling of these regulatory interactions has been observed in a number of cancers (Ruggero, 2013).

Eukaryotic translation initiation begins with the cooperative assembly of the 43S preinitiation complex (PIC), composed of the eIF2/GTP/Met-tRNA_i_ ternary complex (TC), eIF1, eIF1A, eIF5, and eIF3 on the 40S ribosomal subunit. In canonical eukaryotic translation, the 43S PIC recruits mRNAs by engaging the eIF4F cap-binding complex to form the 48S PIC. Within the 48S PIC, eIF1, eIF1A, eIF3, and eIF4G promote the accurate scanning of the mRNA leader region (Hinnebusch, 2011, Hinnebusch, 2014) and ensure the proper recognition and pairing of the start codon with Met-tRNA_i_.

**Figure 1:**
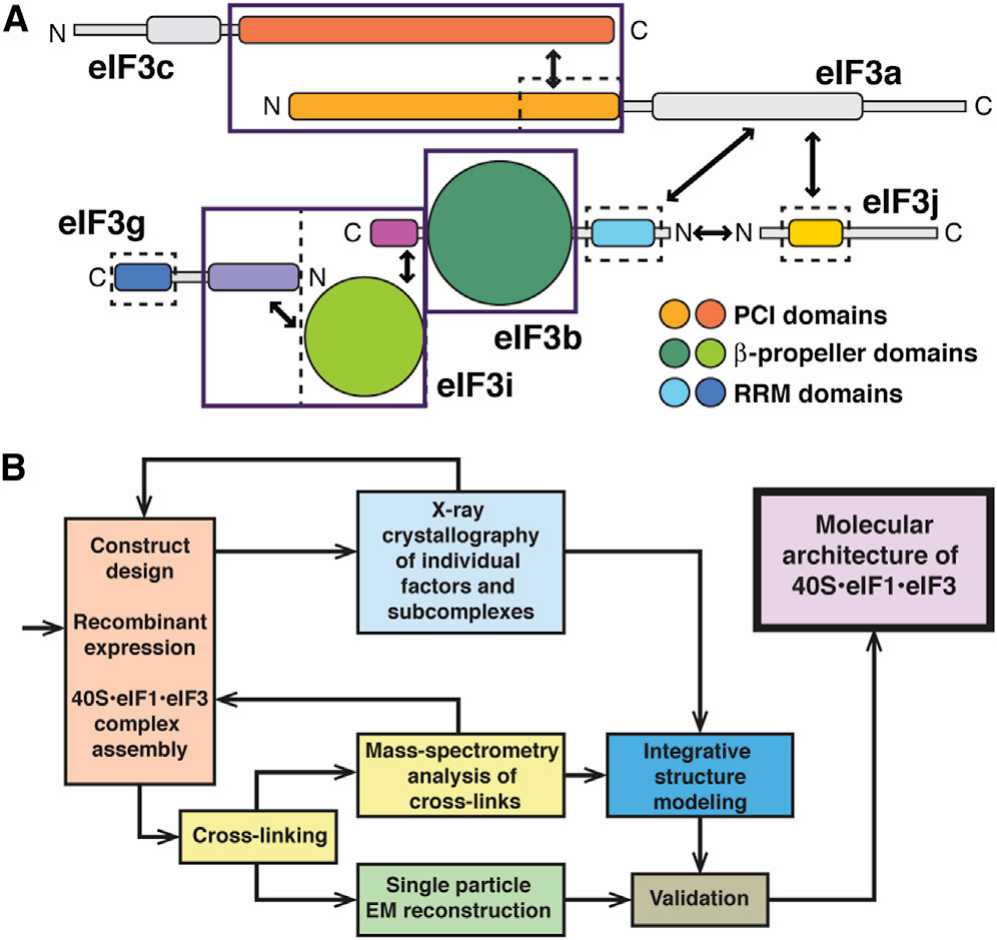
Domain Organization of *S. cerevisiae* eIF3 and Experimental Approach (A) Domain map of the six subunits of *S. cerevisiae* eIF3. Structured domains are shown as rectangles or spheres and are individually colored. Domains with known structural motifs are designated in the legend. Predicted unstructured regions are shown as thin gray lines. Known eIF3 interactions are indicated by arrows. Modules whose crystal structures are described in this paper are boxed, whereas previously described structures are indicated by narrow dashed lines. (B) Schematic outline of the hybrid experimental approach, highlighting the techniques used in this study.

EIF3 is a large and structurally complex molecular assembly that, in the majority of eukaryotes, consists of 11–13 subunits (eIF3a-eIF3m) with a molecular weight of 600–800 kDa (Hinnebusch, 2006, Valásek, 2012). Six of the subunits (eIF3a, eIF3c, eIF3e, eIF3k, eIF3l, and eIF3m) contain PCI (*p*roteosome, *C*OP9/signalosome, e*I*F3) modules, and two (eIF3f and eIF3h) contain MPN (*M*pr1-*P*ad1-*N*-terminal) domains. The PCI⋅MPN core is structurally conserved in the 26S proteasome lid, the COP9 signalosome, and eIF3, forming a distinctive, multilobed structure (Enchev et al., 2010). PCI modules are characterized by an N-terminal helical domain (HD) and a C-terminal winged helix domain (WHD) (Ellisdon and Stewart, 2012), which mediates PCI dimerization (Ellisdon et al., 2012). In the subnanometer EM structures of the proteasome lid, six WHDs oligomerize to form a horseshoe-shaped arc with their HDs radiating outward (Beck et al., 2012, Lander et al., 2012), an organization also observed in the EM reconstructions of eIF3, the 43S, and 43S⋅IRES complexes and the COP9 signalosome (Enchev et al., 2010, Enchev et al., 2012, Hashem et al., 2013a, Hashem et al., 2013b, Querol-Audi et al., 2013). *S. cerevisiae* and related yeasts lack six components of the PCI⋅MPN core, retaining only two PCI proteins among the six universally conserved eIF3 core subunits (eIF3a/Tif32, eIF3b/Prt1, eIF3c/Nip1, eIF3i/Tif34, eIF3g/Tif35, and eIF3j/Hcr1) (Figure 1A). The conserved core displays a modular architecture, with the interaction between eIF3b and the C-terminal portion of eIF3a connecting the PCI subunits to the eIF3g/eIF3i subcomplex and to eIF3j (Zhou et al., 2008).

Recent EM reconstructions as well as in vivo and in vitro experiments indicate that the N-terminal ends of the helical subdomains of the eIF3a/eIF3c PCI modules form two intermolecular bridges with the 40S subunit in the vicinity of ribosomal proteins rpS13/uS15 and rpS27/eS27 as well as rpS26/eS26, rpS1/eS1, and rpS0/uS2 (Hashem et al., 2013a, Querol-Audi et al., 2013, Valásek et al., 2003). In addition, footprinting and hydroxyl-radical probing experiments implicate helix 16 of the 40S subunit in eIF3 binding (Pisarev et al., 2008). Other studies have placed eIF3j, which interacts with the eIF3b-RRM (RNA recognition motif) and the eIF3a-CTD (C-terminal domain) (Chiu et al., 2010, ElAntak et al., 2007, Fraser et al., 2004, Nielsen et al., 2006), near the decoding center of the 40S subunit (Fraser et al., 2007). Nevertheless, information about the molecular architecture of eIF3 is incomplete due to the lack of atomic structures of eIF3 *s*ubunits and the dynamic nature of the 40S⋅eIF3 interaction. In this study, we present X-ray structures of all major structured domains of *S. cerevisiae* eIF3 and utilize a combination of negative-stain electron microscopy (EM), chemical crosslinking followed by mass spectrometry (CX-MS) and integrative structural modeling to derive a detailed molecular architecture of the yeast 40S⋅eIF1⋅eIF3 complex.

## Results

### Overall Structures of the Full-Length eIF3a and eIF3c PCI Modules and the eIF3a/eIF3c Heterodimer

**Figure 2:**
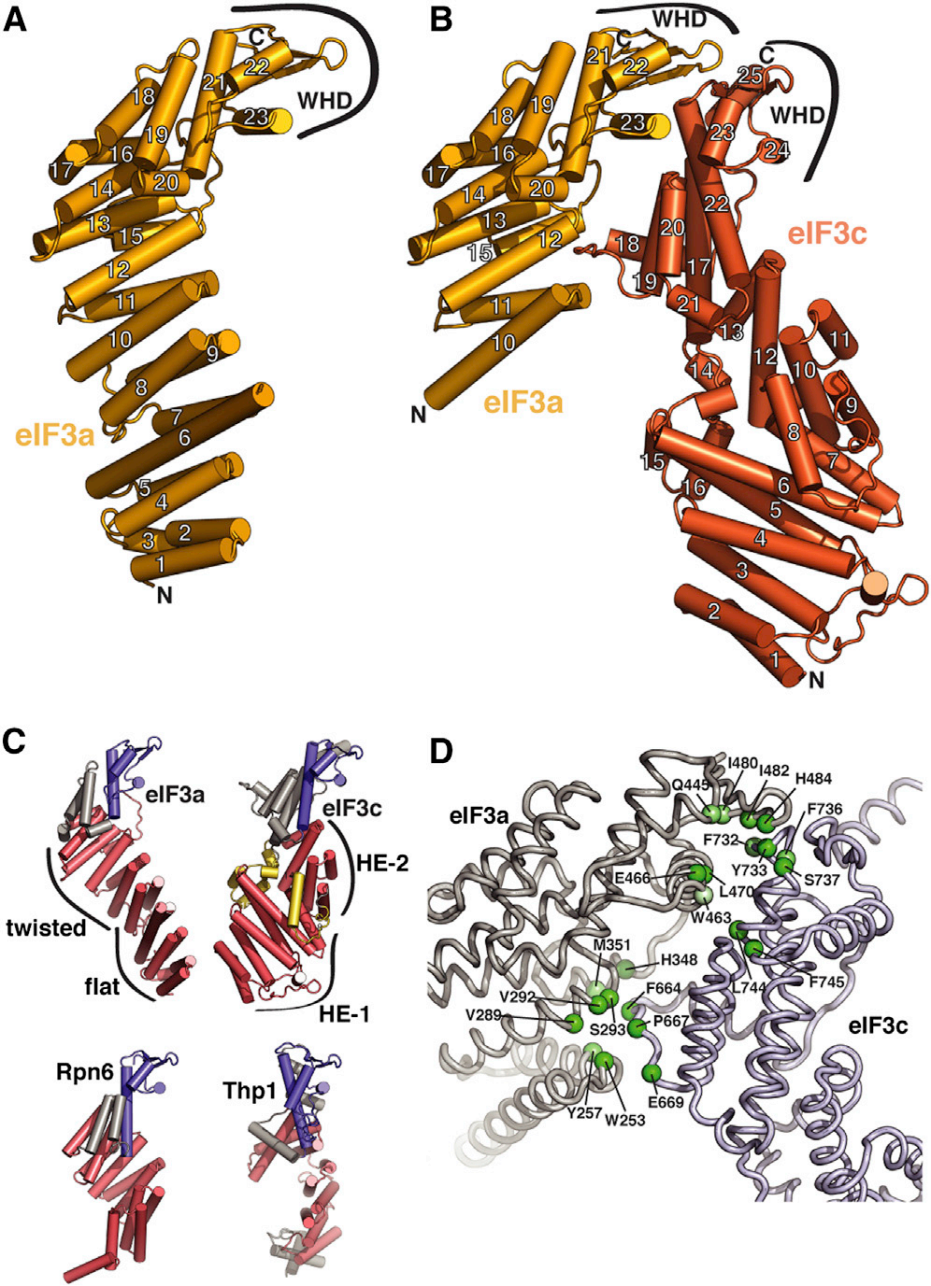
Structures and Interactions of *S. cerevisiae* eIF3 PCI-Domain Proteins (A) Cartoon representation of the crystal structure of the full PCI domain of eIF3a (chain A) with numbered helices. (B) Cartoon representation of the crystal structure of the eIF3a/eIF3c PCI dimer, colored as in Figure 1A and with numbered helices (with the exception of short 3/10 helices). (C) Comparison of PCI helical domains. The crystal structures of eIF3a (chain A) and eIF3c were aligned by superposition of their WHDs (colored blue) to each other and to Rpn6 (PDB 3TXN, Pathare et al., 2012) and Thp1 (PDB 3T5V-chain B, Ellisdon and Stewart, 2012), previously solved PCI proteins with extended helical domains (shown in red). Features of the eIF3a and eIF3c helical regions are labeled, and elements within eIF3c that stabilize the kink in the helical domain are shown in yellow. (D) Detail of the eIF3a/eIF3c interface. Conserved residues within the two interaction regions observed in the crystal structure are shown as spheres and labeled. Stronger green shading signifies a higher degree of conservation.

As a first step in our hybrid approach (Figure 1B), we obtained a number of soluble eIF3 fragments and subcomplexes that were amenable to crystallographic analysis (Table S1 available online). The structure of the full-length PCI domain of eIF3a (*S. cer.* residues 1–496) was solved by single-wavelength anomalous dispersion (SAD) with selenomethionine (Se-Met)-labeled protein to a resolution of 3.3 Å (Table S2). As observed in a truncated structure of eIF3a and in previously characterized PCI modules (Ellisdon and Stewart, 2012, Khoshnevis et al., 2014), the WHD is structurally similar to other PCI proteins, whereas the N-terminal domain has a series of distinct, flatly arranged helical repeats (Figure 2A). The positioning of these helices is reminiscent of the concertina-like arrangement of tetratricopeptide repeats (TPR), although, as in other PCI proteins, TPR-like sequence elements are not detectable. Unlike other PCI HDs, which typically have a significant right-handed superhelical twist, eIF3a has two distinct segments to its repeat. Whereas helices 10–14 have a right-handed pitch, helices 1–9 are arranged along a flat plane that is distinct from other PCI proteins with extended helical repeats (Figure 2C).

Using the full eIF3a PCI-domain structure as a guide, N-terminal eIF3a truncations were generated to obtain crystals of a dimeric eIF3a/eIF3c assembly, encompassing the full predicted PCI domain of eIF3c (*S. cer.* residues 251–812) and residues 228–496 of eIF3a. The structure of the dimeric eIF3a/eIF3c complex was solved at 3.5 Å resolution using Se-Met SAD (Figure 2B and Table S2). The superposition of the common eIF3a WHD component in these structures provides a detailed atomic model for the full eIF3a/eIF3c PCI heterodimer. As expected, the PCI domain of eIF3c shares the basic architecture of the fold (Figure 2B) but with a more elaborate helical domain that extends over 450 residues (Figure 2C). The helical domain can be subdivided into two regions: helical-element 1 (HE-1) is composed of helices 1–7, whereas helical element 2 (HE-2) contains helices 9–13. HE-1 and HE-2 form TPR-like right-handed superhelices similar to those found in other PCI proteins, with the relative orientations of HE-1 and HE-2 defined by helix 8, which disrupts the helical repeat at the HE-1/HE-2 interface, inducing an almost 90° bend in the domain (Figures 2B and 2C). An extensive, conserved protein segment formed by a 40 amino acid insertion between helices 13 and 17 meanders back into the HE-1/HE-2 interface to further stabilize the kink (Figure 2C).

**Figure S1:**
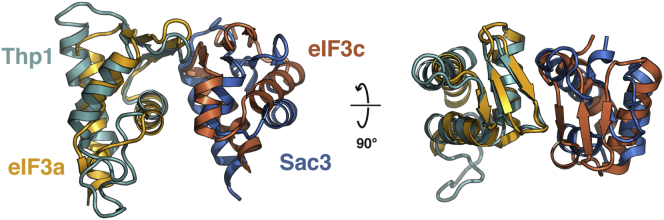
Comparison of WHD Interactions in eIF3a/eIF3c and Thp1/Sac3, Related to Figure 2 The WHDs of Thp1 and eIF3a were superposed, aligning their recognition helices. The positions of their partner proteins are offset by 6 Å, indicating a high degree of variability in WHD interactions within PCI-domain assemblies.

The dimerization interface between eIF3a and eIF3c involves two distinct interaction regions. Helix 23 in the WHD of eIF3a engages the WHD of eIF3c (Figures 2B and 2D) in an arrangement similar to the Thp1-Sac3 PCI-dimer (Ellisdon et al., 2012). Because the WHD domains are the least well-ordered regions in our structure, the details of the interaction cannot be unambiguously established but involve a number of conserved hydrophobic side chains on eIF3a (W463, L470, I480, and I482) and eIF3c (F732, Y733, F736, L744, and F745) as well as a number of conserved polar and charged residues (Q445, E466, and H484 on eIF3a and S737 on eIF3c) (Figure 2D). A comparison between eIF3a⋅eIF3c and Thp1⋅Sac3 shows that, although the overall interaction mode is conserved between these two PCI complexes, the relative positions of the WHDs differ by up to 6 Å between these two heterodimers (Figure S1). The second interaction interface between eIF3a and eIF3c involves a loop between helices 18 and 19 of eIF3c that engages a cleft formed by helices 11, 12, and 15 of eIF3a (Figures 2B and 2D), allowing a stacking interaction between residue F664 of eIF3c and Y257 of eIF3a. This interaction induces a rearrangement of up to 3 Å at the end of eIF3a helix 15 to accommodate the eIF3c loop.

### Implications for Understanding Mammalian eIF3 Architecture and eIF3a/eIF3c⋅HCV/CSFV IRES Interactions

**Figure 3:**
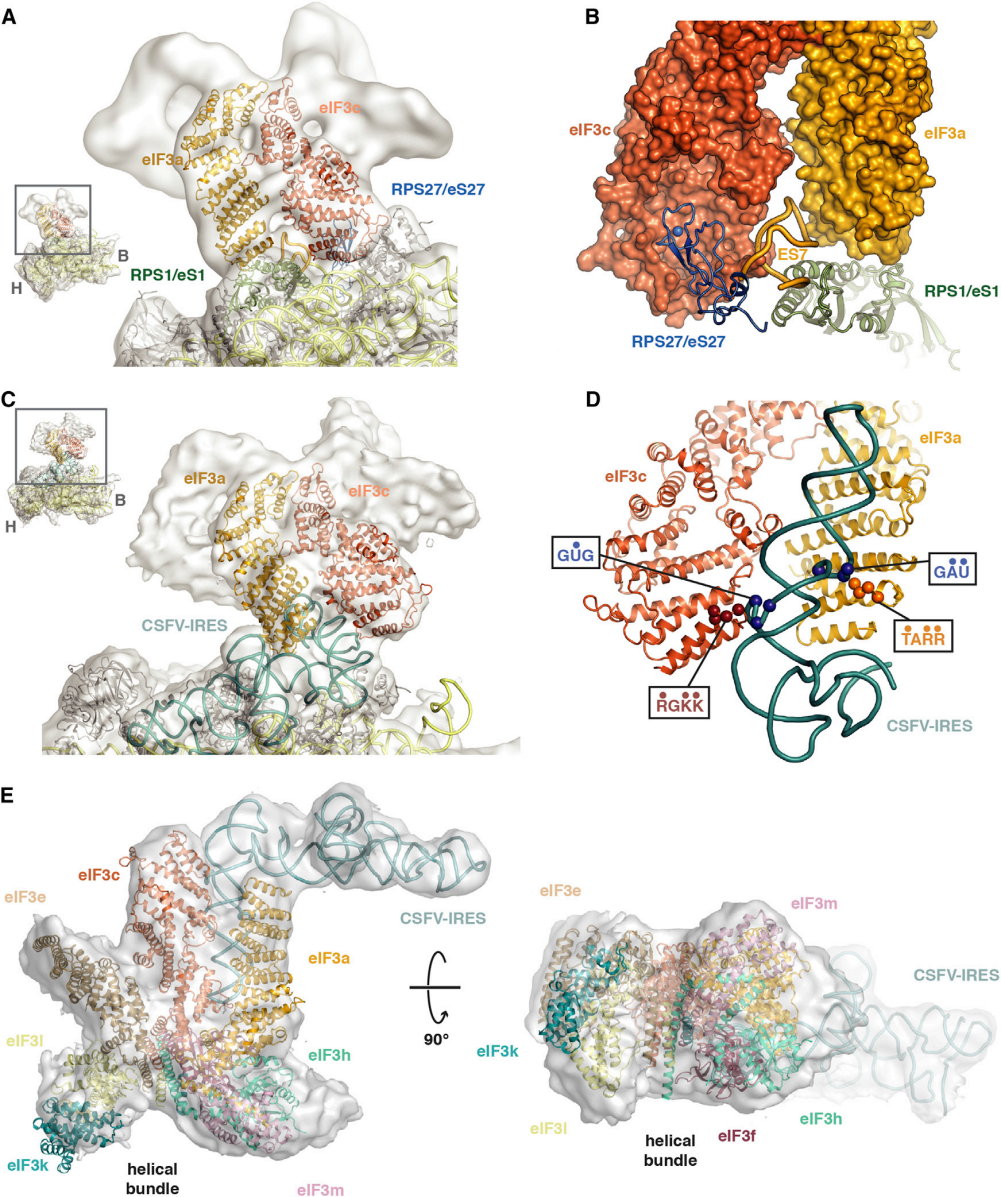
Docking of eIF3a/eIF3c in the PCI⋅MPN Core Density of Mammalian 43S and 43S⋅IRES EM Maps (A) Rigid-body fitting of the eIF3a/eIF3c PCI heterodimer model into the EM density of the 43S EM map. EIF3a and eIF3c are labeled and colored as in Figure 1A. Ribosomal proteins rpS1/eS1 (green) and rpS27/eS27 (blue) from the docked yeast 40S structure (Ben-Shem et al., 2011) are highlighted. Other ribosomal proteins are shown in gray and the ribosomal RNA in yellow. (B) Detail of the position of the eIF3a/eIF3c helical domains after rigid-body fitting and their proposed interaction with ribosomal proteins rpS1/eS1 (green), rpS27/eS27 (blue), and RNA expansion element ES7 (orange). (C) Rigid-body fitting of the eIF3a/eIF3c PCI heterodimer model into the 43S⋅IRES EM map. (D) Detail of the interaction between the CSFV-IRES and the eIF3a/eIF3c dimer in the docked structure. Residues of eIF3a and eIF3c important for IRES binding are shown as spheres and identified by their sequences. The dots indicate positions mutated in the equivalent human complex (Sun et al., 2013). RNA elements protected by eIF3 in IRES domain IIIB are shown as blue spheres and are identified by their sequences. The blue dots indicate extrahelical bases. (E) Model of the complete PCI⋅MPN core of eIF3 (based on currently available crystal structures and models) (Figure S2A) docked into the 43S⋅IRES EM map (Hashem et al., 2013b).

Yeast eIF3 has a significantly reduced repertoire of eIF3 components, but the conservation of eIF3a and eIF3c in all eukaryotes is high. Docking of the eIF3a/eIF3c PCI heterodimer into the EM density of the mammalian 43S (Hashem et al., 2013a) reveals an excellent fit into the two structural elements that mediate 40S binding by the PCI-MPN core of eIF3 (Figure 3A). In this placement, eIF3c engages the Zn-binding knuckle of rpS27/eS27 through a conserved pocket within HE-1, whereas eIF3a primarily contacts rpS1/eS1 (Figure 3B). The two PCI modules flank ES7, the only expansion segment within the central RNA domain that defines the 40S platform (Figure 3B).

Many viral internal ribosome entry site (IRES)-containing RNA elements require the presence of eIF3. The recent structure of the 40S-bound CSFV IRES in complex with eIF3 revealed the structural basis of this interaction, with the IRES RNA occupying the eIF3 binding site at the 40S platform and creating a secondary binding site for the PCI⋅MPN core (Hashem et al., 2013b). Because IRES interactions are also mediated by eIF3a/eIF3c, we were able to model the interaction by fitting the eIF3a/eIF3c heterodimer into the 43S⋅IRES EM density map without any adjustments (Figure 3C). This fit of the eIF3a/eIF3c heterodimer places two loop elements (between helices 2 and 3 of eIF3a and helices 1 and 2 of eIF3c) important for IRES and 43S binding (Sun et al., 2013) in close contact to two extrahelical bulges conserved within domain IIIB of the CSFV and HCV IRESs (Figure 3D). Consistent with our model, these RNA elements are protected from RNase digestion when bound by eIF3 (Sizova et al., 1998, Sun et al., 2013).

**Figure S2:**
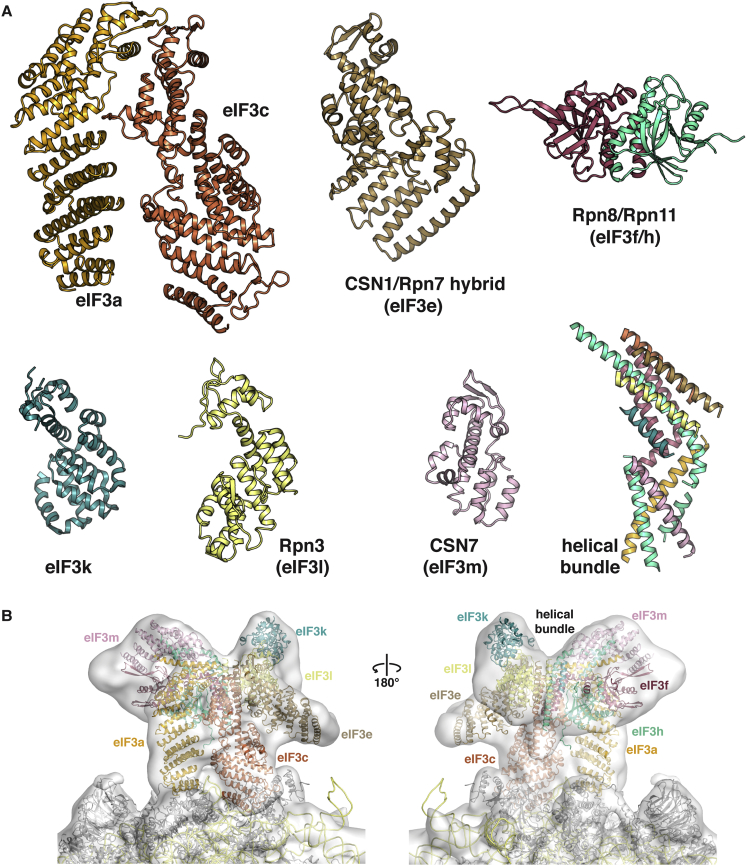
Individual Structures Used to Assemble the Mammalian eIF3 PCI⋅MPN Core, Related to Figure 3 The PCI-MPN model was assembled from the eIF3a/eIF3c PCI heterodimer presented in this paper, a hybrid of the helical domain of the partial CSN1 structure (PDB 4LCT) (Lee et al., 2013), with the missing WHD replaced by the rpS7 model from 26S EM structure (PDB 4B4T) (Beck et al., 2012) and the Rpn8/Rpn11 dimer model (PDB 4O8X) (Pathare et al., 2014) used to represent eIF3f/h. Additional models were the mammalian eIF3k structure (PDB 1RZ4) (Wei et al., 2004), the EM-fitted model of Rpn3 (PDB 4B4T) (Beck et al., 2012) and the structure of CSN7 (PDB 3CHM) (Dessau et al., 2008). Finally, the model for the helical bundle was derived from the model for the proteasome bundle (PDB 3J47) (Estrin et al., 2013).

Based on the convincing fit of the eIF3a/eIF3c structure into the EM density, we attempted to dock the recently solved paralogs of eIF3e (CSN1) (Lee et al., 2013) and the eIF3h-eIF3f heterodimer (Rpn8/Rpn11) (Pathare et al., 2014, Worden et al., 2014) into the eIF3⋅IRES EM density along with older models for eIF3k, eIF3m, and eIF3l (Figure S2A). Using the previously identified positions of the various eIF3 subunits as a guide (Querol-Audi et al., 2013), we fitted the models to agree with features of the EM density (Figure 3E). The proposed arrangement defines the molecular interactions within the core subunits, with the HCV⋅IRES, and with the 40S subunit. Remarkably, even the helical bundle structure recently proposed for the 26S proteasome lid (Estrin et al., 2013) fits the remaining density after placement of the PCI and MPN domains (Figure 3E), suggesting that it is a conserved feature of the PCI⋅MPN core of these functionally diverse complexes. The final model also fits the lower-resolution mammalian 43S EM maps (Figure S2B).

### Structure of the eIF3b β-Propeller Domain

**Figure 4:**
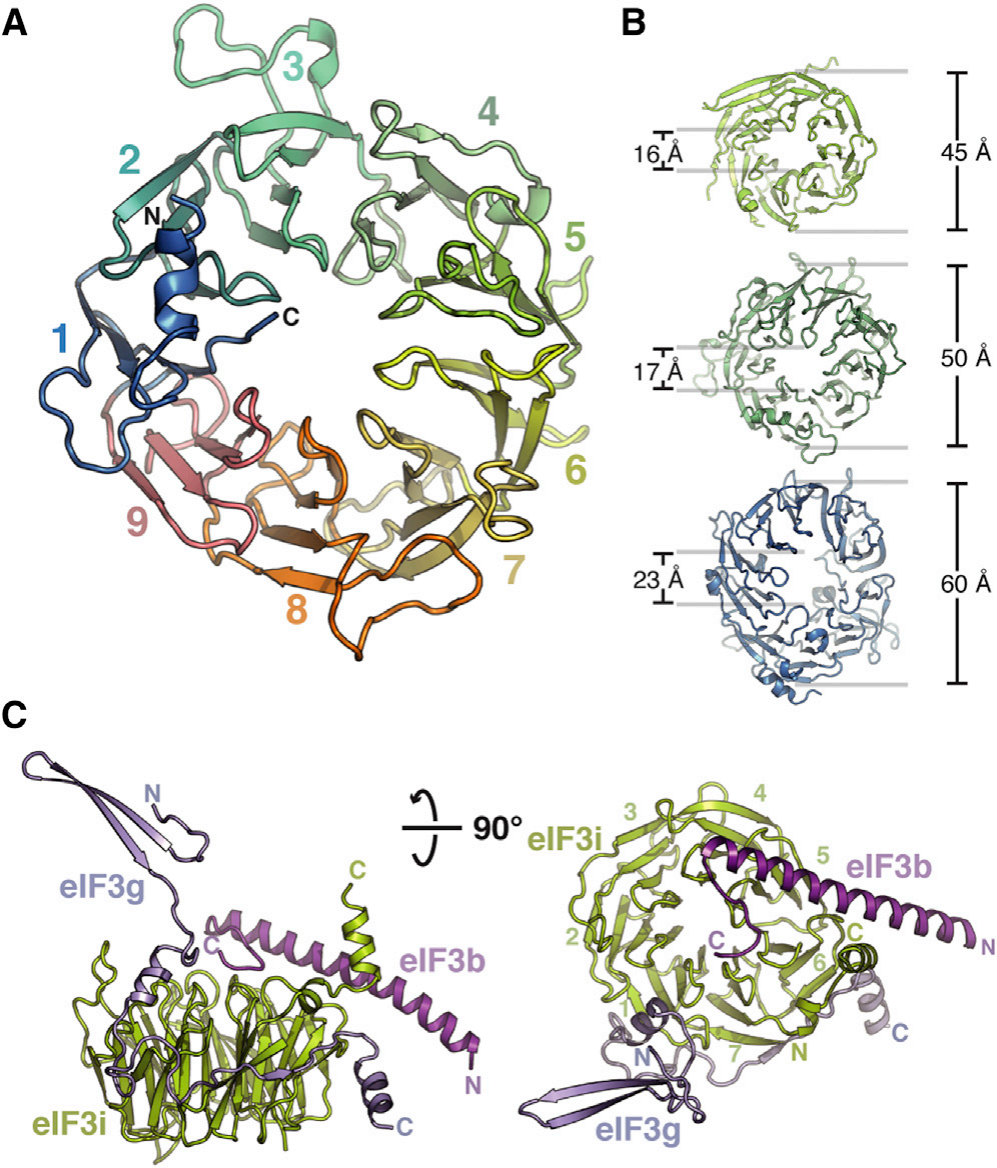
Structures of the eIF3b β Propeller and of the eIF3b-CTD/eIF3i/eIF3g-NTD Complex (A) Cartoon representation of the crystal structure of the middle domain of eIF3b (monomer A, unswapped). Individual blades are colored and labeled, and the position of the N and C termini are indicated. (B) Comparison of the β propeller diameters and pore dimensions among eight- (PDB 1R5M, Cerna and Wilson, 2005), nine-, and ten-bladed (PDB 3F6K, Quistgaard et al., 2009) β propellers. (C) Cartoon representation of the crystal structure of the eIF3b-CTD/eIF3i/eIF3g-NTD trimer, colored as in Figure 1A.

**Figure S3:**
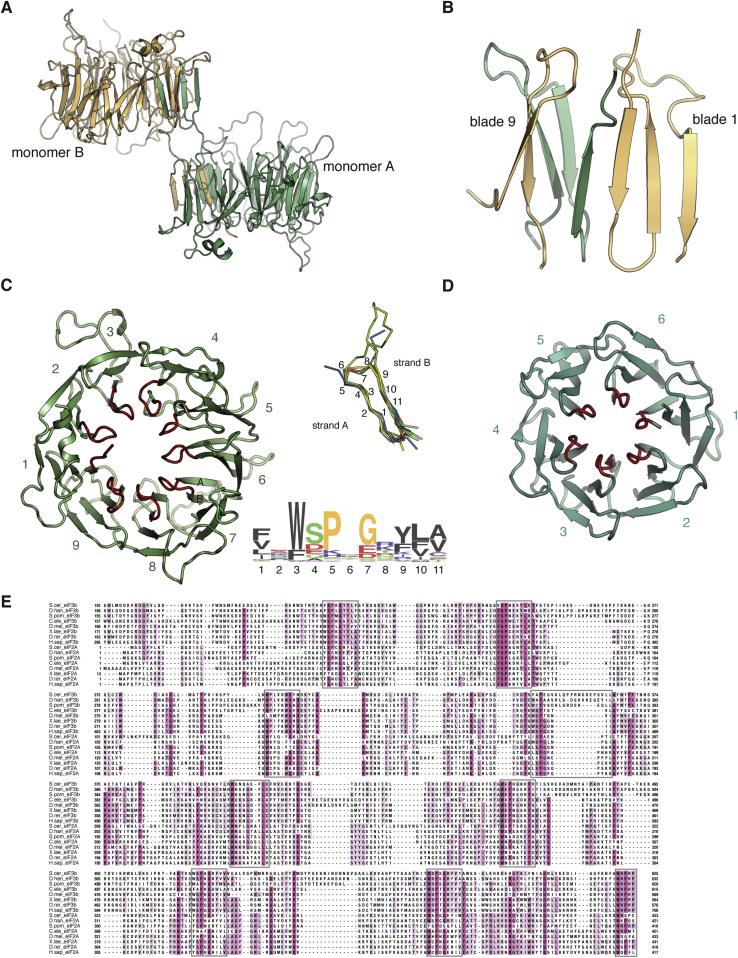
EIF3b β-Propeller Domain Swap, Conserved Structural Motif, and Alignment with eIF2A, Related to Figure 4 (A) Domain swap in the eIF3b β-propeller structure. (B) Detail of the swap - three strands from the invading domain complete the sheets in blades 1 and 9. (C) Conserved motif in the A-B loop region of individual eIF3b β-propeller blades. Inset - Superposition of all AB strands and LOGO depiction of conservation within a large number of eIF3b sequences. (D) Structure of TolB, which shares a similar motif in its linkages. (E) Multiple sequence alignment of eIF3b and eIF2A β-propeller domains, showing conserved residues in magenta. eIF2A shares the same number of blades and the conserved sequence motif (boxed) with eIF3b.

The eIF3b subunit plays a critical anchoring role within the 5 subunit core of eIF3. Though structural information is available for the N-terminal RRM domain (ElAntak et al., 2007, Khoshnevis et al., 2010) and a segment of the C-terminal tail (Herrmannová et al., 2012), great uncertainty remained about the central region of the protein, predicted to form either a single or a double β-propeller structure. We purified and crystallized the 57 kDa central domain of eIF3b (*S. cer.* residues 132–626) and solved its structure using Se-Met SAD to a resolution of 2.2 Å (Table S3). The middle domain of eIF3b forms a β propeller with nine blades, a configuration not previously observed in a native polypeptide (Figure 4A). In our crystals, two β propellers are present in the asymmetric unit, with a domain swap encompassing segments of blades 1 and 9 (Figures S3A and S3B). For clarity, our other figures depict a monomeric, unswapped eIF3b model.

The middle domain of eIF3b begins with a short helical element, followed by the first blade of the β propeller. The A strand of this blade is provided by the most C-terminal strand in a 3+1-type velcro closure. The blades of eIF3b are fairly symmetric, with extensive loops decorating blades 3, 4, 5, 7, and 8 (Figure 4A). The central channel of eIF3b is marked by a conserved structural loop motif that constricts the “bottom” entrance of the central cavity, creating a funnel-like shape (Figure S3C). The structure and underlying sequences of the loops are conserved among the various blades of *S. cerevisiae* eIF3b and in the blades of eIF3b β propellers from other organisms (Figures S3C and S3E). This particular loop orientation is also seen in the six-bladed TolB β propeller, which has a similar consensus sequence (the AxSPD motif) linking strands A and B (Figure S3D) (Abergel et al., 1999, Chen et al., 2011). The nine-bladed eIF3b β propeller fills the gap between previously characterized eight- and ten-bladed β propellers (Figure 4B). A search for other nine-bladed β propellers in the *S. cerevisiae* genome revealed that the conserved sequence element and its associated nine blades are also present in eIF2A (Figure S3E), a repressor of IRES-mediated translation initiation that is downregulated during stress events in *S. cerevisiae* (Kim et al., 2011). It remains to be investigated whether these shared structural features reflect functional commonalities with eIF3b.

### Structure of the Trimeric eIF3b-CTD/eIF3i/eIF3g-NTD Complex

**Figure S4:**
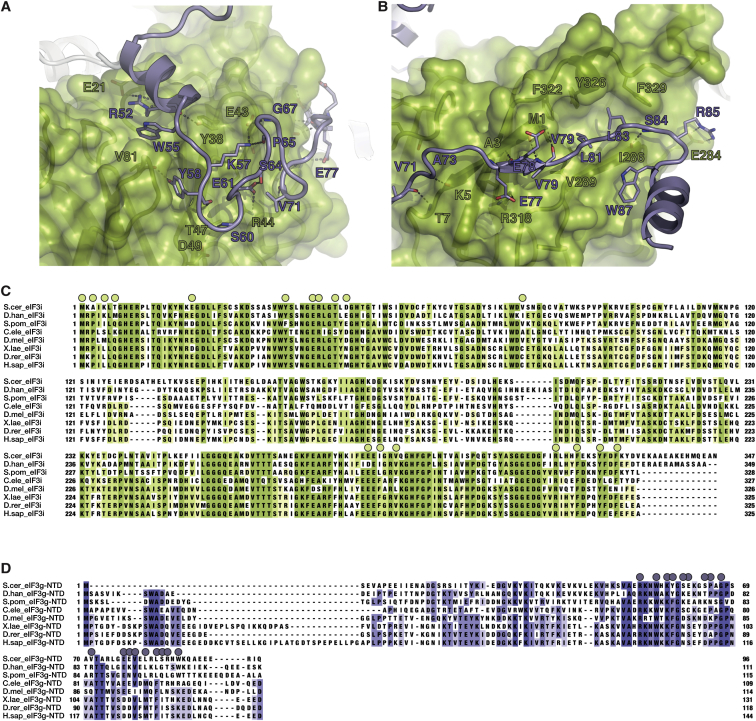
Details and Residues Conservation of eIF3i/eIF3g-NTD Interaction Surface, Related to Figure 4 (A–D) Details of the (A) first and (B) additional two conserved interaction elements between eIF3i (green) and eIF3g (purple). Residues of eIF3g and eIF3i involved in direct contacts are shown as sticks and labeled in green (eIF3i) or purple (eIF3g). Representative sequences from 40-organism alignments of (C) eIF3i and (D) the eIF3g-NTD, colored by percent conservation. Residues labeled in panels (A) and (B) are marked by spheres.

The C-terminal helical domain of eIF3b mediates the association of eIF3b with the dimeric eIF3i/eIF3g complex (Herrmannová et al., 2012). Crystals were obtained for a complex containing the eIF3b-CTD (*S. cer.* residues 655–698), full-length eIF3i, and the eIF3g-NTD (N-terminal domain) (*S. cer.* residues 1–135). The heterotrimeric structure was solved to a resolution of 2 Å using molecular replacement with the eIF3b-CTD/eIF3i complex as the search model (Table S3) (Herrmannová et al., 2012). The arrangement of eIF3i and the eIF3b fragment was nearly identical to the previously solved dimeric complex, with a root-mean-square deviation (rmsd) of 0.4 Å. Of the 135 residues in the crystallized eIF3g fragment, clear density was observed for the first 96 residues. The first 46 residues of eIF3g form a β hairpin that makes an important crystal contact but does not directly interact with eIF3i or eIF3b. Indeed, no residue of the eIF3g-NTD makes a direct contact with the eIF3b-CTD (Figure 4C). Residues 47–90 of eIF3g are responsible for the tight association with eIF3i, meandering along one-third of the outside surface of the eIF3i β propeller and making extensive contacts with blades 1, 6, and 7 of eIF3i (Figures 4C, S4A, and S4B). The molecular interaction between the eIF3g-NTD and eIF3i can be divided into three areas. The first is defined by the insertion of three eIF3g residues (R52, W55, and Y58) between blades 1 and 2 of eIF3i and involves both hydrophobic and polar interactions (Figure S4A). The second is characterized by extensive polar contacts along the outside of blades 1 and 7, including a short β strand (residues E77–V79) extending the sheet of blade 7 of eIF3i (Figure S4B). Finally, another set of exclusively hydrophobic interactions (residues L81, L83, and W87 of eIF3g) occurs between blades 6 and 7 of eIF3i (Figure S4B). The most prominent elements in the interaction surface are highly conserved among all organisms (Figures S4C and S4D), suggesting that the interaction mode between eIF3i and eIF3g is universally conserved.

### Single-Particle Reconstructions of 40S⋅eIF1⋅eIF3 Particles from *Lachancea kluyverii*

**Figure 5:**
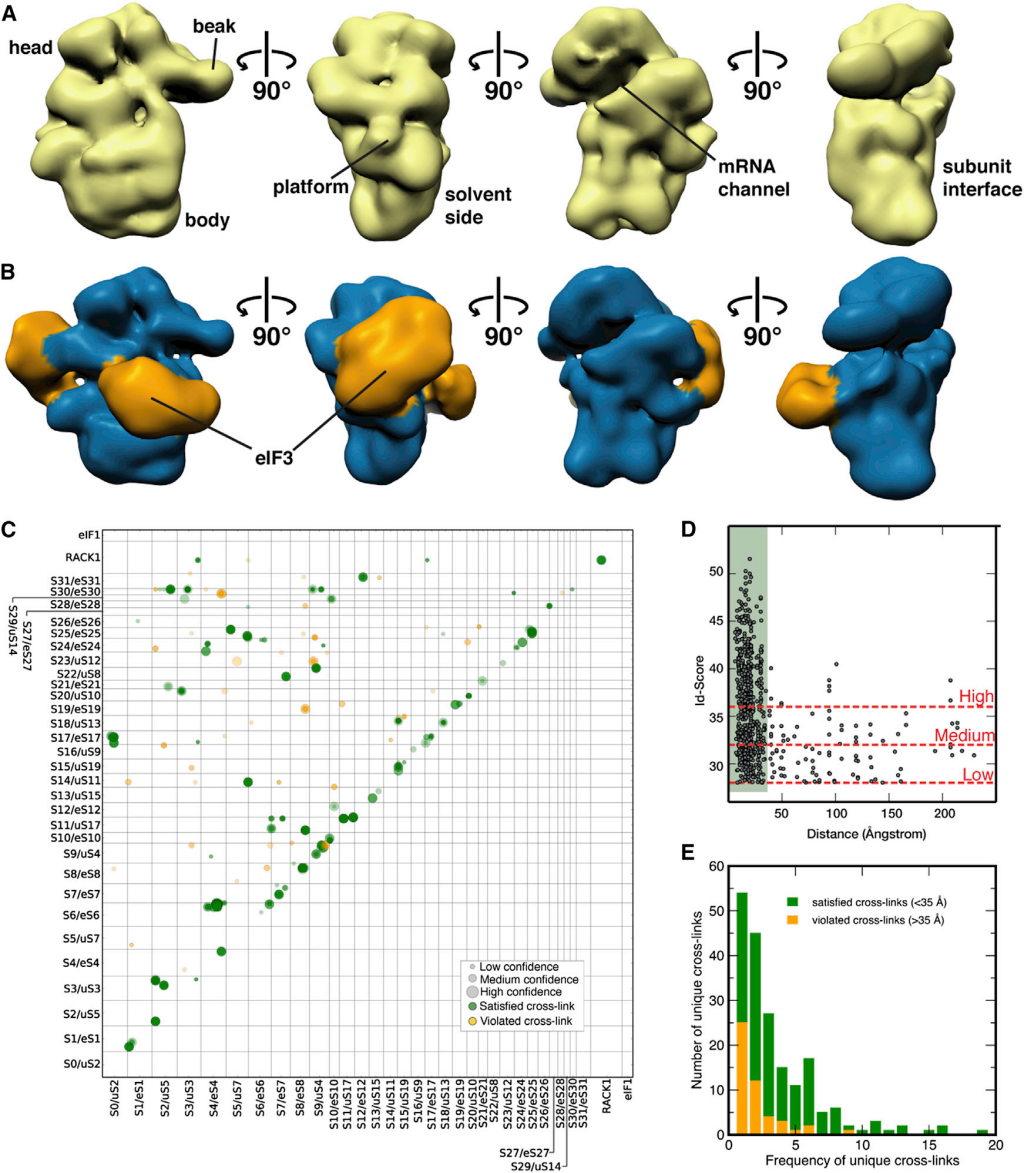
Single-Particle Reconstruction and CX-MS Analysis of the Yeast 40S⋅eIF1⋅eIF3 Complex (A and B) Views of EM reconstructions of the (A) unoccupied and (B) occupied fractions of the *L. kluyveri* 40S⋅eIF1⋅eIF3 complex sample with labeled ribosomal landmarks. (C) Matrix of all unique crosslinks between and within subunits of 40S from CX-MS analyses of multiple 40S⋅eIF1⋅eIF3 samples. Crosslinks were mapped onto the X-ray structure of the yeast 40S particle and were colored in green (crosslink distance <35 Å) or orange (crosslink distance >35 Å). The size of the circle for each mapped crosslink is proportional to its Id score. Multiple identifications of a particular crosslink are indicated by a stronger color intensity due to overlaid circles. (D) Scatter plot of mapped crosslink distances against Id score of the 40S data set. Crosslinks are grouped into three classes of similar size (high = Id score >36; medium = 32 < Id score < 36; and low = 28 < Id score < 32). Satisfied crosslinks (distance <35 Å) are within the green background. (E) Frequency distribution (as defined in Table S4) of detected unique crosslinked residues within the 40S matrix. Satisfied and violated crosslinks are colored as in (C).

Electron microscopy studies of initiation events have been hampered by the labile nature of 40S-eIF1 interactions during cryoelectron microscopy (cryo-EM) sample preparation as described for both yeast and mammalian 40S⋅eIF1 complexes (Hashem et al., 2013a, Passmore et al., 2007). Because eIF1 is critical for eIF3 binding to 40S, we similarly observe no consistent eIF3 occupancy in 40S samples prepared for cryo-EM, even with samples crosslinked by GraFix (Kastner et al., 2008). In contrast, identical samples prepared by negative stain show clear density for eIF3, suggesting that this preparation method is more compatible with a functional 40S⋅eIF1⋅eIF3 assembly. The 28 Å single-particle reconstruction of the *L. kluyveri* 40S⋅eIF1⋅eIF3 complex (Figure 5B) reveals two large areas of extra density on the solvent-exposed side of 40S compared to complexes without eIF3 (Figure 5A). Similar to the eIF3 density in the mammalian 43S structure, one extra density is localized below the platform, matching the eIF3a/eIF3c PCI heterodimer in size and shape, whereas the other region of extra density is on the solvent-exposed side of the 40S subunit, halfway between the platform and the beak. In our hybrid structural approach, we use the EM envelope as a boundary to validate the results of our integrative structure modeling outlined below.

### Crosslink Mass Spectrometry Analysis of the 40S⋅eIF1⋅eIF3 Complex

We carried out CX-MS experiments to identify contact points between eIF3 subunits and 40S⋅eIF1 and to guide our placement of eIF3 subunits on 40S. Because of the propensity of *S. cerevisiae* eIF3 samples to form dimers due to the blade swapping within the eIF3b subunit (Khoshnevis et al., 2012) (Figure S3A), we carried out the CX-MS experiments on eIF3 samples from the budding yeasts *Lachancea kluyveri* and *Debaryomyces hansenii* (see the Extended Experimental Procedures for details). These organisms, like *S. cerevisiae*, only possess the universally conserved, six-component eIF3.

To define the position of eIF3 on 40S, we prepared and crosslinked samples of the yeast eIF3 core bound to 40S⋅eIF1. Samples were either crosslinked in high salt to prevent nonspecific ribosome binding and eIF3 aggregation or, alternatively, in low salt but with an additional sucrose gradient ultracentrifugation step to remove dimeric 40S/40S⋅eIF3 species and unbound eIF3. Samples were subjected to LC-MS/MS analysis, yielding 965 interlinks and intralinks (i.e., crosslinks between different proteins or within the same protein, respectively) (Table S4). Specifically, we were able to detect 155 interlinks between eIF3 subunits and 40S and 461 interlinks between 40S ribosomal proteins.

**Figure S5:**
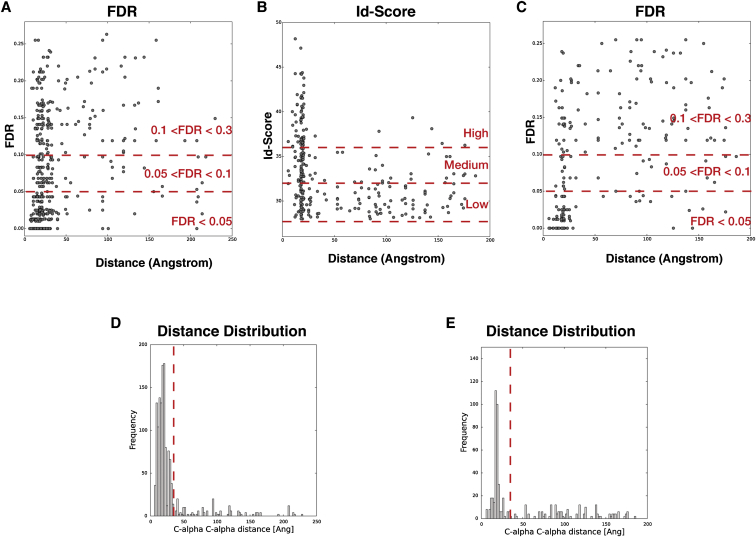
Scatter Plots and Histograms of Crosslink Data, Related to Figure 5 (A) Scatter plot of mapped cross-link distances against the FDR rate for the 40S data set. Cross-links are grouped into three classes of roughly similar size. For FDR assignment into FDR < 0.05; 0.05 < FDR < 0.1 and 0.1 < FDR < 0.3; for Id-Score assessment cross-links were divided into High = Id-Score > 36; Medium = 32 < Id-Score < 36 and Low = 28 < Id-Score < 32. (B) Scatter plot of distances versus the Id-Score for interlinks of eIF3 with the 40S⋅eIF1 particle and links between and within different subunits of eIF3. Distances in this case were calculated average distances for the final set of solutions from our integrative modeling approach. (C) Distances of the same eIF3 data set plotted against the FDR rate. (D) Histogram of mapped distances of all cross-linked residues within the 40S data set versus their frequency. The 35 Å threshold is indicated as a red dotted line. It can be clearly seen that the vast majority of cross-linked residues spans a distances smaller than 35 Å and that those cross-linked residues bridging a larger distance follow no obvious pattern. (E) Histogram of distances against frequency for all cross-linked residues of eIF3 with the 40S⋅eIF1 particle and linked residues between and within different subunits of eIF3. In this case, we calculated average distances for the final set of solutions from our integrative modeling approach. The histogram shows a trend similar to the 40S data set.

The extensive number of crosslinks connecting 40S ribosomal proteins permitted a detailed analysis of the characteristics of our data set. When mapped onto the X-ray structure of the *S. cerevisiae* 40S subunit (Ben-Shem et al., 2011), 86% of these crosslinks fall within 35 Å (Figure 5C and Table S5), the maximal lysine Cα-Cα distance that our crosslinker can bridge (Leitner et al., 2012). Within this crosslink test set, the distribution of all mapped distances versus the Id score (Figure 5D) (Rinner et al., 2008) or versus the FDR rate (Figure S5A) (Walzthoeni et al., 2012) shows the expected correlation, as 96% of crosslinks with an Id score of >36 and 94% crosslinks with an FDR of <0.05 are satisfied (Table S5). However, this analysis also showed that a majority of crosslinks in the lower confidence score ranges are also satisfied, suggesting that useful information is lost if standard data cutoffs are applied. Another characteristic observed in our test data set is that certain unique crosslinks are detected more frequently across independent samples than others (Figure 5C and Table S4). These highly redundant crosslinks were more likely to be satisfied than those detected once or only a few times (Figure 5E), suggesting that the number of occurrences of a specific crosslink should be an important consideration when analyzing our data set.

### Integrative Structural Modeling of the 40S⋅eIF1⋅eIF3 Complex Architecture

**Figure S6:**
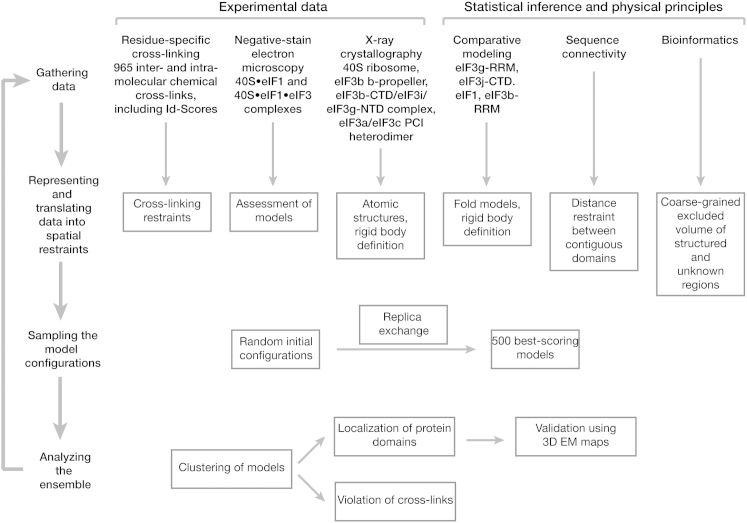
Schematic of the Integrative Modeling Strategy, Related to Figure 6 Schematic of the integrative modeling approach highlighting the individual data inputs and the four stages in our modeling strategy.

**Figure 6:**
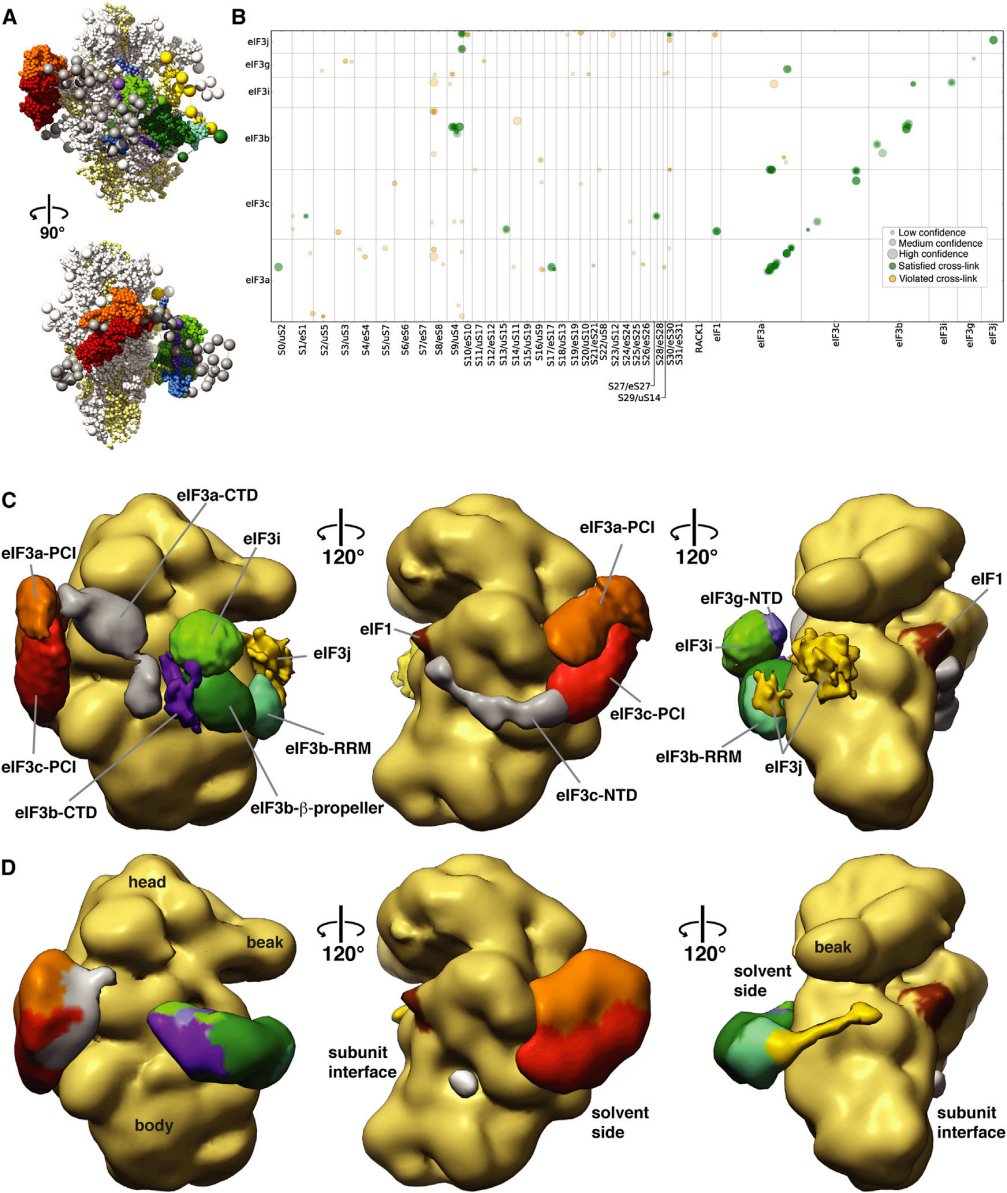
Integrative Modeling of the 40S⋅eIF1⋅eIF3 Complex (A) Representative example of one of our modeling solutions, showing the bead models used in our calculations. Domains of eIF3 are colored as in Figure 1A. (B) Matrix of unique crosslinks between eIF3 and the 40S⋅eIF1 particle and within subunits of eIF3. Intensity, size, and color code of crosslinked residues are as in Figure 5C. (C) Localization densities for eIF3 domains superposed on the unoccupied 40S EM reconstruction. The localization densities for each domain are contoured at 1.6 times their estimated molecular volumes. EIF3 domains are colored as in Figure 1A, and linker regions are labeled and colored gray. The position of eIF1 is indicated in brown. (D) Difference density of the occupied and unoccupied 40S⋅eIF1⋅eIF3 EM structures. The density is colored according to the positions of eIF3 domains given by the localization densities shown in (C).

**Figure S7:**
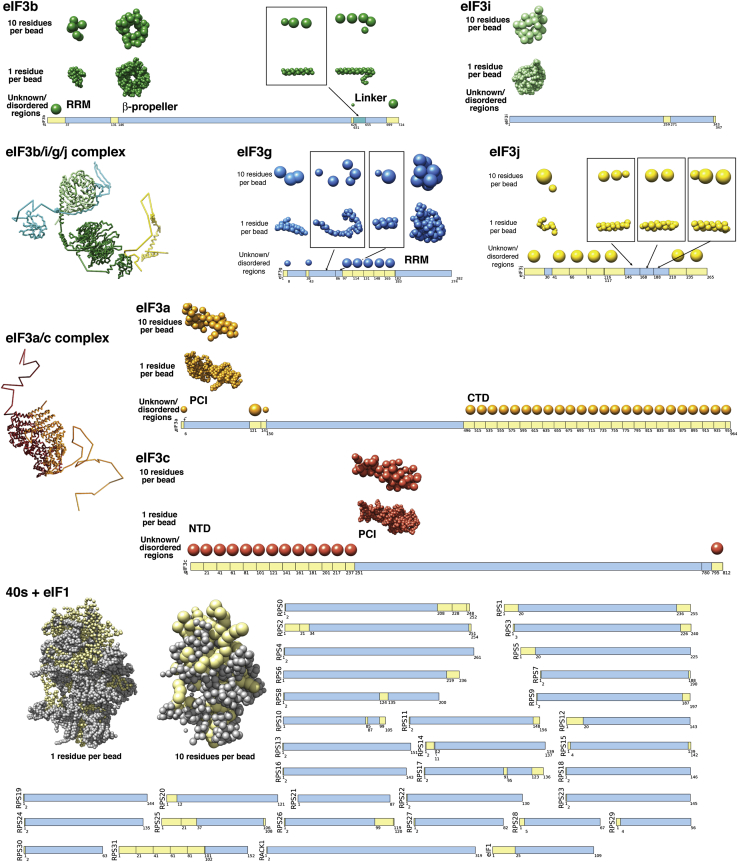
Representation of the 40S⋅eIF1⋅eIF3 Subunits, Related to Figure 6 All subunits (eIF3b, eIF3i, eIF3j, eIF3g, eIF3a, eIF3c and 40S⋅eIF1) are represented by a set of beads, arranged into either rigid bodies corresponding to structured parts (blue segments in the bars, e.g., eIF3b β propeller) or flexible strings corresponding to unknown/disordered parts (yellow segments in the bars, e.g., eIF3c-NTD or eIF3a-CTD). The low resolution (10 residues per beads) and high resolution (1 residue per bead) representations are constrained together in the same rigid body. Furthermore, three interfaces are kept rigid (eIF3a/eIF3c PCI; eIF3b(655-699)/eIF3i/eIF3g(43-86); eiF3j(30-41)/eIF3b-RRM) as well as the structured parts of 40S⋅eIF1. In summary, the three resulting complexes (eIF3b/i/g/j, eIF3a/c and 40S⋅eIF1) are made of rigid domains and interfaces, connected by flexible linkers.

The observed characteristics of the 40S crosslink test set influenced our strategy for the integrative structural modeling of the 40S⋅eIF1⋅eIF3 complex (Figure S6). In addition to promoting crosslinks with high redundancy, we decided to include all crosslinks with an Id score greater than 28 (FDR <0.3) and used the information gained from the test set to split the crosslink data set into three confidence classes (high, medium, and low; Figures 5D, S5, and Table S5). In order to fully explore the contributions of high-, medium-, and low-confidence crosslinks, we opted for a Bayesian framework to allow us to qualify every piece of information based on the test set. The goal was to lessen the impact of inconsistent crosslinks while benefitting from accurate crosslinks exclusively present within the low and medium confidence ranges (Figure 6B and Experimental Procedures). The 965 crosslinks from the 40S⋅eIF1⋅eIF3 data set were used to model the positions and orientations of the six eIF3 subunits on the 40S⋅eIF1 complex, using crystallographic structures and comparative models of the 40S subunit, eIF1 protein, eIF3 domains, and eIF3 interfaces (Figures 6A, S6, and S7, and Extended Experimental Procedures). 40S, eIF1, and eIF3 domains were represented by sets of beads arranged into either rigid bodies or flexible strings (Figures 6A and S7). None of the available EM maps were integrated in the calculation. Candidate models were ranked by a scoring function (described in the Extended Experimental Procedures) that reflected how well the models satisfied crosslinking data as well as excluded volume and sequence connectivity restraints (Russel et al., 2012).

**Figure S8:**
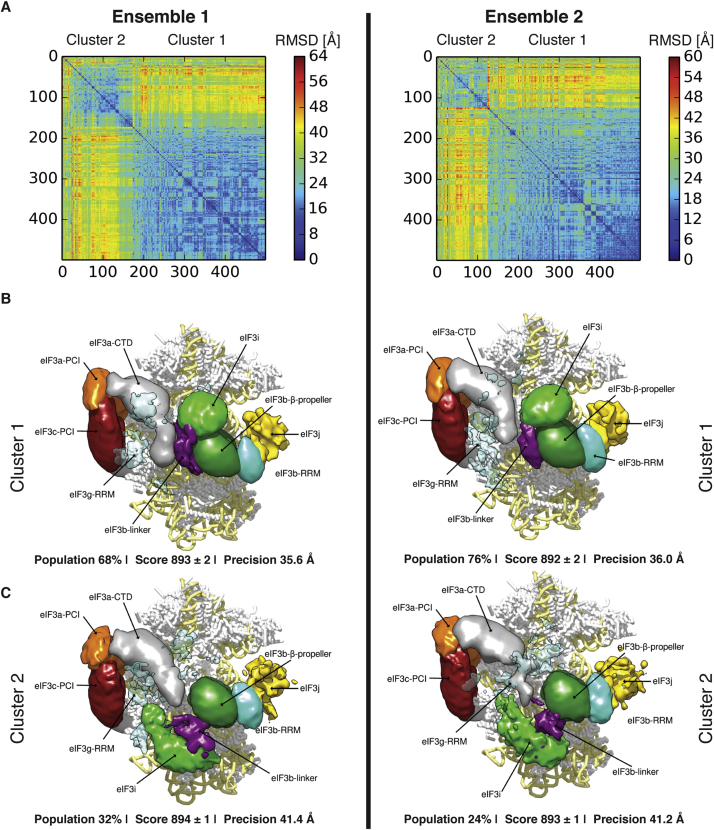
Localization Densities of the Ensemble of Solutions, Related to Figure 6 (A–C) The 180,000 sampled models were divided into two ensembles (left and right columns) and independently analyzed to assess the sampling convergence. The matrix of the rmsd (A) between all pairs of the 500 best-scoring models (solutions) indicates the presence of two clusters of similar structures. The color scale corresponds to the rmsd distance between structures in Å. The localization densities of cluster 1 (B) and cluster 2 (C) are contoured such that each volume represents 1.6 times the molecular volume of the corresponding domain. Cluster 1 and cluster 2 differ in the position of eIF3i. The precision values are calculated over all eIF3 subunits.

We computed 90,000 structural models by sampling the positions and orientations of the rigid bodies as well as the positions of the remaining beads, guided by the scoring function. The 500 best-scoring models were grouped into two clusters, using the rmsd as a structural similarity criterion (Figure S8 and Extended Experimental Procedures). The cluster of models with the higher population and lower average score was chosen as the final solution set.

These solutions satisfied the excluded volume of the beads and their sequence connectivity restraints. As expected from our experimental design, most of the satisfied crosslinks within our data set of 126 unique eIF3-eIF3 and 40S⋅eIF1-eIF3 crosslinking restraints were redundant and of high confidence (Figures 6B and S5 and Table S5). Importantly, the eIF3-eIF3 and 40S⋅eIF1-eIF3 data set had crosslink confidence and frequency distributions comparable to the 40S⋅eIF1 crosslink data set (Table S5 and Figure S5).

The average pairwise rmsd of the solutions in the favored cluster is 36 Å, or 30 Å if the poorly determined eIF3g-RRM domain is omitted from the rmsd calculation. This precision allowed us to determine the positions and orientations of the eIF3 domains (Table S6). We represented the cluster of solutions by individual localization densities (Figures 6C and S8), defined as the probability of observing a specific eIF3 domain at a given point in space. Our modeling results, as described by these localization densities, place eIF3 at the back of 40S, arranged into a continuous structure that encircles the 40S⋅eIF1 complex, comprising two large modules and three linker regions (Figure 6C). These results are fully validated by the EM reconstructions, as our localization densities are in remarkable agreement with our difference density EM map (Figure 6D). In fact, the extensive overlap between the localization densities and the EM map allowed us to assign specific eIF3 domains to regions of the EM difference density (Figure 6D).

### EIF3 Subunit Placement on 40S and Comparison between Mammalian and Yeast 40S⋅eIF3 Complexes

**Figure 7:**
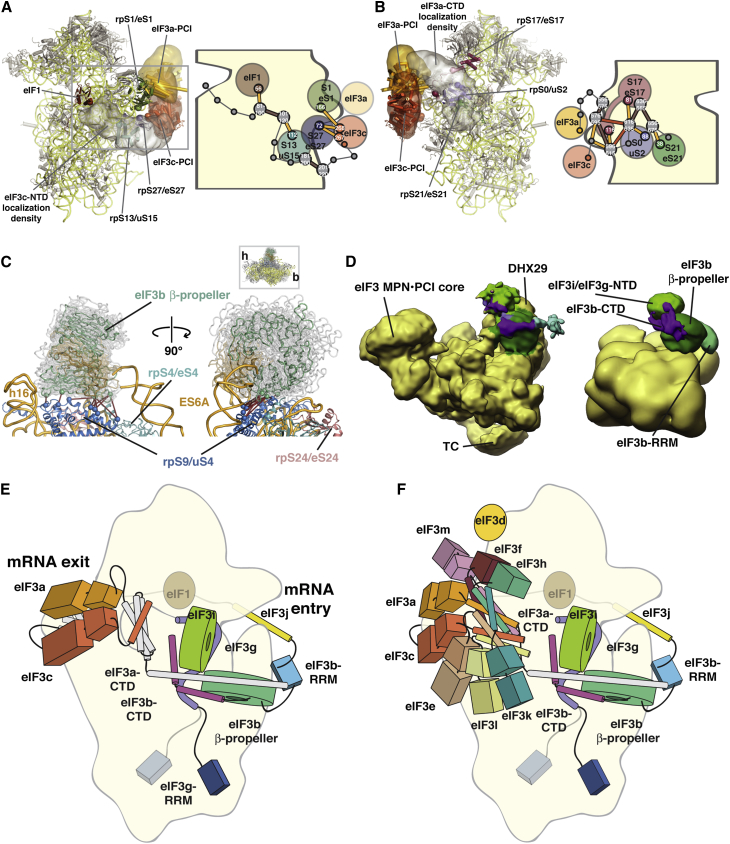
Placement and Interactions of eIF3 Components on 40S (A) (Left) Cartoon depiction of the 40S⋅eIF1 structure model, showing ribosomal proteins in gray and RNA in yellow. Proteins that are crosslinked to eIF3c are highlighted and labeled. The localization densities for the eIF3a/eIF3c PCI heterodimer and the eIF3c-NTD are shown as transparent densities and are colored as in Figure 1A. (Right) Schematic depiction of the crosslinking pattern of the eIF3c-NTD. The bead model of the eIF3c-NTD is shown as gray spheres linked by thin gray lines and is labeled with the residue range it represents. Interlinks are shown in gold and intralinks in red. (B) Same as (A) but showing the localization density for the eIF3a-CTD and highlighting the inter- and intralink network that helps define its orientation. (C) Molecular model of the 40S-bound eIF3b β-propeller domain. 40S RNA and protein elements in the vicinity of the binding site are individually colored and labeled. Ten representative eIF3b structures are shown as gray ribbons, with an average structure colored as in Figure 1A. Residues involved in crosslinks are shown as spheres, and crosslinks are in red. (D–F) (D) Comparison of the mammalian 43S EM map (left) and the localization densities (superposed on the unoccupied yeast EM map) for the eIF3b/eIF3i/eIF3g complex (right). Domains are colored as in Figure 1A and are labeled. Model of the consensus positions of various eIF3 elements in the yeast (E) and mammalian (F) 40S⋅eIF1⋅eIF3 complexes, integrating the information from our hybrid approach with known interactions of eIF3 elements to derive a comprehensive model of the molecular architecture of eIF3 on the 40S subunit. Individual eIF3 components are labeled and colored as in Figures 1A and 3E. The position of the eIF3d subunit is based on extra density observed in the mammalian 43S EM structure.

The localizations of the eIF3b β propeller, the eIF3b-RRM, and the PCI modules of eIF3a and eIF3c have a precision of <30 Å and are the best-defined components within our modeling solutions (Table S6 and Figure 6C). The high precision of the eIF3a/eIF3c PCI heterodimer localization stems, in part, from three unique crosslinks between our structural model of the eIF3a/eIF3c PCI heterodimer and 40S (Figures 6B, 7A, and 7B). However, an extensive network of crosslinks involving the peripheral, non-PCI regions of eIF3a (*S. cer.* residues 496–964) and eIF3c (*S. cer.* residues 1–251) provide critical additional restraints (Figure 6B). These linking elements connect the PCI modules with the eIF3b/eIF3i/eIF3g module and with eIF1, eIF2, and eIF5, respectively. Because no structural information is available for these regions, they are represented by beads comprising 20 residue segments in our modeling (Figure S7). Despite this coarse representation, eIF3c-NTD and eIF3a-CTD form tightly clustered interaction networks containing both inter- and intralinks that provide additional restraints on either side of the eIF3a/eIF3c PCI heterodimer-binding site (Figures 7A and 7B). The localization densities for the linker regions match regions of weak difference density in our EM reconstructions (Figures 6D, 7A, and 7B), further suggesting that portions of the eIF3c-NTD and eIF3a-CTD form small ordered regions. In particular, the extensive crosslink network involving the first part of the eIF3a-CTD and the C-terminal helix of eIF3c hints at the formation of a structure analogous to the helical bundle present in the larger PCI⋅MPN core (Figures 7B, 7E, and 7F). Biochemical evidence suggests that the second half of the eIF3a-CTD extends near the entrance of the mRNA channel, interacting with h16/h18, rpS2/uS5, rpS3/uS3, and the eIF3b-RRM (Valásek et al., 2001, Valásek et al., 2003). Although we do not observe any specific crosslinks that support this orientation, an extended orientation for this region of eIF3a is consistent with our overall model.

In our solutions, the eIF3b β propeller localizes between RNA helices ES6A and h16 and above rpS9/uS4, rpS4/eS4, and rpS24/eS24 with a high precision of 15 Å (Table S6). The placement is guided by ten unique crosslinks between four eIF3b residues and seven rpS9 residues (Figures 6B and 7C) and is consistent with previous RNase protection experiments that mapped an interaction between mammalian eIF3 and h16 (Pisarev et al., 2008). To obtain a representative set of structures from our solutions, we further clustered the eIF3b β propeller conformations on the 40S subunit using an rmsd cutoff of 3.5 Å (Extended Experimental Procedures). This analysis allowed us to select ten structures from our solutions that represent the most frequent positions of the eIF3b β propeller. The overlay of the selected eIF3b β-propeller structures reveals a tight cluster of structures that share a common interaction surface with 40S (Figure 7C). The placements of the eIF3b-CTD/eIF3i/eIF3g-NTD and of the eIF3b-RRM are less precise, relying exclusively on crosslinks and sequence connectivity constraints to the eIF3b β-propeller domain, as there are no satisfied crosslinks with 40S (Figure 6B). Indeed, the position of the eIF3b-CTD/eIF3i/eIF3g-NTD complex is the feature that defines the two clusters within our 500 best-scoring solutions. The position of our best-solution set relies heavily on a redundant, medium-confidence crosslink between eIF3i and the eIF3b β propeller, whereas the position in the less-populated cluster is dominated by connectivity restraints that are less constrained in this orientation, leading to the lower precision observed for this cluster (Figure S8 and Table S6). In our final solution set, the position of the eIF3i β propeller within the eIF3b-CTD/eIF3i/eIF3g-NTD subcomplex is consistently oriented orthogonally to the eIF3b β propeller (Figure 6C), with the “bottom” face of the eIF3i β propeller facing the “top” surface of the eIF3b β propeller (Figures 7E and 7F). This arrangement places the C-terminal helices of eIF3b in an elongated conformation along the “bottom” and “top” surfaces of the eIF3b and eIF3i β propellers, respectively, and the eIF3g-NTD is localized between eIF3i and 40S near the interface of the β propellers, consistent with evidence that eIF3g stabilizes the eIF3b/eIF3i interaction (Herrmannová et al., 2012, Khoshnevis et al., 2012) and interacts with ribosomal proteins rpS3/uS3 and rpS20/uS10 (Cuchalová et al., 2010). At the N-terminal end of eIF3b, the localization density of the eIF3b-RRM is adjacent to the eIF3b β propeller near the mRNA entrance channel, linking eIF3b to eIF3j, which our solutions place in its expected position near the mRNA channel entrance (Fraser et al., 2007).

Consistent with our localization densities, the EM envelope of the mammalian 43S complex, despite the presence of the DHX29 helicase (Hashem et al., 2013a), shows features that agree with our model. Of the density elements assigned to eIF3, a toroid-shaped element between the platform and the beak of 40S closely matches our position for the eIF3b β propeller (Figure 7D). Previously, this density was tentatively attributed to eIF3i (Hashem et al., 2013a). Furthermore, the localization densities of the eIF3b-CTD/eIF3i/eIF3g-NTD complex and the eIF3b-RRM match regions of weak additional density in the mammalian 43S structure (Figure 7D), supporting the localization and orientation of the eIF3b/eIF3i/eIF3g module in our solution set. This extensive agreement between the EM densities and our modeling solutions gives us confidence that our models for the yeast and mammalian 40S⋅eIF1⋅eIF3 complexes reveal the regions of the 40S subunit involved in eIF3 interactions as well as the positions and relative orientations of nearly all eIF3 domains and the likely paths of all major linker domains (Figures 7E and 7F).

## Discussion

The eIF3 complex plays a prominent role in regulating the intricate molecular events of translation initiation. Its large size and modular structure have made molecular studies of this assembly challenging, and a comprehensive molecular understanding of the 40S⋅eIF3 interaction has remained elusive.

Our hybrid approach allowed for a large number of low-resolution restraints to be effectively integrated and optimized, resulting in a sophisticated and surprisingly detailed interaction map of eIF3 on the ribosome (Figures 7E and 7F). This arrangement enables eIF3 components to completely encircle the 40S subunit and to engage and coordinate the binding of other components of the translation initiation machinery at both ends of the mRNA channel. EIF3 can therefore act as a scaffold, ensuring that factors required at both the mRNA channel entry and exit are assembled simultaneously, allowing eIF3 to act as the “orchestra conductor” (Valásek, 2012) during initiation, coordinating the input from a number of “sections” to ensure that mRNA recruiting and scanning at one end occurs only when elements critical for AUG recognition and scanning modulation are assembled at the opposite end. Interestingly, because eIF1 and eIF3j have high 40S-binding affinities, interactions at the ends of the two eIF3 “arms” may enable the cooperative assembly of the full 43S PIC.

This model is consistent with the vast majority of biochemical and genetic data that has accumulated over the years (Hinnebusch, 2006, Hinnebusch, 2014, Marintchev and Wagner, 2004, Valásek, 2012). Mutations and disruptions in factors mapped to the mRNA channel entrance result in a leaky scanning phenotype and defects in rescanning downstream of short uORF sequences (Chiu et al., 2010, Elantak et al., 2010, Nielsen et al., 2006, Cuchalová et al., 2010), suggesting that eIF3b, eIF3g, eIF3i, and eIF3j prevent AUG codon bypass and modulate rescanning downstream of uORFs by engaging the mRNA in the vicinity of the channel entrance. At the other end of the eIF3 “clamp,” the eIF3c-NTD extends across the subunit interface, connecting the PCI⋅MPN core of eIF3 with the mRNA channel and making critical contacts with eIF1, eIF5, and TC and, in mammals, with eIF4 to create an assembly that is highly proficient at codon differentiation during 5′ UTR scanning (Karásková et al., 2012). In contrast to mutations in the eIF3b/eIF3i/eIF3g/eIF3j module, mutations in the N-terminal tail of eIF3c result in defects in TC recruitment and scanning fidelity, suggesting distinct roles for the two “arms” of eIF3 that extend into the mRNA channel (Phan et al., 2001, Valásek et al., 2004).

The interactions mediated by the six universally conserved eIF3 subunits probably represent the core functions of eIF3 during translation initiation. The remaining six PCI⋅MPN eIF3 components not found in budding yeasts are likely to act as “gatekeepers,” engaging elements within mRNPs (including the cap-binding complex) to regulate their access to the ribosome. In *S. cerevisiae*, the loss of eIF3 PCI⋅MPN core components correlates with the simplified 5′ UTR structures of most transcripts and the low intron density found in these organisms (Hinnebusch, 2014). Our detailed molecular description of the interaction between eIF3 and the 40S ribosomal subunit maps individual functionalities of eIF3 to distinct regions of the 43S PIC and provides an intricate structural framework to integrate biochemical, biophysical, and genetic studies of eukaryotic translation initiation.

## Experimental Procedures

### Cloning, Purification, and Complex Assembly

A full list of constructs used in this study is given in Table S1. The purification protocols for eIF1, various eIF3 subunits and complexes, and the 40S subunit are described in Extended Experimental Procedures.

### Crystallization, Data Collection, and Structure Determination

All proteins (15–20 mg/ml) were crystallized by sitting-drop vapor diffusion. Native and Se-Met SAD data were collected at beamline PX of the Swiss Light Source and processed in XDS (Kabsch, 2010). Structures were solved using AutoSol or Phaser MR in Phenix (Adams et al., 2010) and models built using Phenix AutoBuild (Adams et al., 2010) and COOT (Emsley et al., 2010). Model refinement was performed with Phenix (Adams et al., 2010). More details are provided in the Extended Experimental Procedures and Tables S2 and S3.

### Chemical Crosslinking Coupled to Mass Spectrometry (CX-MS)

EIF3 samples were assembled from purified, recombinant subunits and were subsequently mixed with 40S subunits as described in the Extended Experimental Procedures. Final 40S⋅eIF1⋅eIF3 samples were assembled under various buffer conditions and crosslinked. Some samples were purified on sucrose gradients before RNase treatment, enzymatic digestion, and enrichment of crosslinked peptides was carried out. LC-MS/MS analysis was performed on an Orbitrap Elite mass spectrometer, and the data were searched using the xQuest/xProphet software pipeline. Further details are provided in the Extended Experimental Procedures.

### Electron Microscopy

The *Lachancea kluyveri* 40S⋅eIF1⋅eIF3 complex was prepared and crosslinked as described for CX-MS. The sample was then subjected to gradient fixation using GraFix (Kastner et al., 2008), grids were prepared by negative staining using uranyl acetate, and images were acquired in a Tecnai F20 electron microscope under low-dose conditions. Single-particle images were split into initiation factor occupied and unoccupied ribosomal complexes based on an initial reconstruction of the whole data set using supervised classification (Imagic Spider RRR). The resolution of the final structure of the initiation factor occupied and unoccupied ribosomal complex was estimated as 28.1 and 24.6 Å, respectively (Fourier shell = 0.5 criterion, semi-independent data half sets).

### Integrative Modeling

The integrative modeling approach proceeded through four steps, as described in Extended Experimental Procedures. To benefit from the entire crosslink data set, the Bayesian scoring function separately modeled the uncertainty of high-, medium-, and low-confidence crosslinks (as defined by Id score and FDR), thereby modulating their relative weights (Rieping et al., 2005). The 275 eIF3-eIF3 and 40S⋅eIF1-eIF3 crosslinks restrained the positions and orientations of eIF3 domains relative to 40S⋅eIF1, whereas the remaining 690 40S⋅eIF1 crosslinks were included as a training set to increase the accuracy of the uncertainty estimation. Because the frequency of crosslink observation across eight independent experiments correlates with their accuracy in our test set (Figure 5E), the unique crosslinks were weighted proportionally to their frequency. An extensive description of the methodology is provided in the Extended Experimental Procedures, and the modeling scripts and models are available at http://salilab.org/40S-eIF1-eIF3.

Extended Experimental Procedures **Reagent Purification, Complex Assembly, and Sample Preparation**

Proteins were cloned into pET-derived vectors using ligation-independent-cloning and expressed in *E. coli* BL21-DE3 pRIL cells in 2xYT media overnight at 18°C. Cells were lysed in 50 mM HEPES pH 7.5, 800 mM KCl, 40 mM imidazole, 0.5 mM Tris-(2-Carboxyethyl) phosphine hydrochloride (TCEP) and 10% Glycerol. *S. cer.* or *L. klu.* His-MBP-tagged eIF3c and His-tagged eIF3a were independently purified over Ni-affinity columns, then combined and purified over a Heparin column. *D. han.* His-eIF3b/eIF3i/eIF3g was expressed from a polycistronic construct and purified over Ni-Sepharose and Heparin columns. Bacterial cells expressing *S. cer.* or *L. klu*. eIF3i were mixed with cells expressing *S. cer.* or *L. klu* His-MBP-tagged eIF3g before lysis and purification over Ni-Sepharose and HiTrap Q. *L. klu.* His-eIF3b, His-MBP-eIF3j and His-eIF1 were expressed separately and purified over Ni-Sepharose and Heparin columns. Samples for crystallography were assembled and digested with TEV protease overnight to remove all tags before final purification over a Superdex 200 gel-filtration column. The 6-subunit eIF3 core complex was assembled from purified *L. klu.* eIF3a/eIF3c, eIF3b, eIF3i/eIF3g (or eIF3b/eIF3i/eIF3g in the case of *D. han.*) and eIF3j before overnight TEV cleavage to remove all tags. After orthogonal purification to remove uncleaved proteins, tags and TEV protease, the sample was applied onto a Superdex 200 column to yield the full eIF3 complex. Se-Met-derivatized proteins were expressed in minimal media supplemented with an amino acid cocktail including Se-Met (Van Duyne et al., 1993).

40S ribosomal subunits were purified from *Lachancea kluyveri* (DSMZ strain 70517). Cells were grown to an OD of 10 in a 50-l fermenter (INFORS) at 24°C, harvested by centrifugation, washed with 1x PBS, resuspended in lysis buffer and snap frozen in liquid nitrogen. Yeast cells were lysed using a cell cracker at 40 kpsi, the lysate cleared by centrifugation (Sorvall SS-34) and then applied onto a high-density sucrose cushion (60% w/v) in low salt for a 24 hr ultracentrifugation at 50k rpm in a TI-70 rotor. Pelleted ribosomal particles were resuspended in high-salt buffer and the subunits separated on 10%–47% w/v sucrose gradients by ultracentrifugation. 40S bands were carefully removed and pooled, then buffer exchanged into 50 mM HEPES pH 7.5, 10 mM MgCl_2_, 75 mM KCl and 0.5 mM TCEP.

Because of the propensity of *S. cer.* eIF3b to form dimers, we focused on two related budding yeasts for our EM and CX-MS experiments. The full 6-component eIF3 complex, lacking only the disordered C terminus of eIF3a (equivalent to *S. cer.* residues 870-964) was assembled from individual components. While we were able to assemble the full eIF3 complex from *Lachancea kluyveri*, we were unable to purify eIF3a and eIF3c from *Debaromyces hansenii*. Because our polycistronic construct for *D. han.* eIF3b/eIF3i/eIF3g expressed large quantities of the trimeric complex, and because of the high degree of conservation among these related budding yeasts, we decided to test whether we could obtain a hybrid complex of *L. klu.* and *D. han.* to increase the variability of the samples for CX-MS. Hybrid eIF3 complexes behaved similarly to the nonhybrid *L. klu.* complex and showed the same areas of extra density in negative-stain single particle reconstructions (data not shown). 40S⋅eIF1⋅eIF3 (10 μM) was assembled with a 1.2x molar excess of initiation factors in 25 mM HEPES, 15 mM MgCl_2_, 125 mM KCl and 2 mM TCEP prior to crosslinking. For each round of experiments, several different salt concentrations (40 mM to 175 mM KCl) as well as sample concentrations (2 μM to 10 μM) were explored to find optimal conditions for stable complex formation prior to crosslinking.

**Crystallization**

All proteins were exchanged into crystallization buffer (20 mM HEPES, 75 mM KCl and 0.5 mM TCEP) prior to crystallization. The eIF3a-PCI domain (18 mg/ml) was crystallized by sitting-drop vapor diffusion against a solution of 14%–18% PEG 3350, 400-600 mM KSCN in a broad pH range (6.8-8.8) using bis-tris-propane, HEPES or bicine as buffers. Crystals belonging to space group C2 grew over 2-5 days and were cryo-stabilized with the addition of 25% ethylene glycol. The eIF3a/eIF3c dimer (20 mg/ml) was crystallized by sitting-drop vapor diffusion against a solution of bis-tris-propane pH 6.0-6.3, 9%–11% PEG 6000 and 400-500 mM KSCN. Crystals belonging to space group P3_2_21 appeared overnight and achieved maximum size after 3 days. Crystals were stabilized and dehydrated over a 2 hr period by the addition of 25% ethylene glycol and 12% PEG 20K over 4 equal concentration steps. This dehydration treatment was essential for diffraction beyond 4.5Å and to promote isomorphicity along the crystallographic c-axis between native and Se-Met crystals. The eIF3b β propeller (20 mg/ml) was crystallized in space group P2_1_ after 2-5 days in 10%–12% PEG MME 5k, 100-200 mM KSCN or KH_2_PO_4_ and pH 8 – 9.5 using Tris-Acetate or CHES as buffers. Crystals were cryo-stabilized with the addition of 25% Glycerol and flash frozen in liquid nitrogen. The trimeric eIF3b-CTD/eIF3i/eIF3g-NTD complex (15 mg/ml) was crystallized in 5%–7% PEG 20K, 25%–28% PEG MME 550 at pH 6.5 with Imidazole or MES buffers and belonged to space group C222_1_. The structures of eIF3a, eIF3a/eIF3c and eIF3b were solved by single-wavelength anomalous dispersion techniques using Se-Met substituted proteins. eIF3b-CTD/eIF3i/eIF3g-NTD was solved using the yeast eIF3b/eIF3i structure (Herrmannová et al., 2012) as a molecular replacement model.

**Structure Determination, Model Building, and Structure Refinement**

The eIF3a, eIF3b β propeller and eIF3b-CTD/eIF3i/eIF3g-NTD structures were solved and built using standard methodologies as described in Experimental Procedures. The structure of the eIF3a/eIF3c heterodimer presented more of a challenge due to its lower resolution, prevalence of split spots and unusually high overall thermal factors (Wilson B = 170 Å^2^; Average refined B factor = 175 Å^2^). Initial models were built into the 3.7 Å experimental SAD maps obtained from AutoSol (Terwilliger et al., 2009), as they were more informative than the phase-extended 3.5 Å maps. After extensive rounds of manual rebuilding in Coot (Emsley et al., 2010) and refinement in Phenix (Adams et al., 2010), eventually incorporating the higher-resolution data, a high-confidence model was built, although the presence of split lattices and the high B-factors prevented further improvement of the R-factors.

**PCI⋅MPN Core Model and EM Fitting**

*H. sapiens* eIF3 subunits were threaded onto the model templates (Figure S2) using Phyre2 (Kelley and Sternberg, 2009). Models are represented as poly-alanine but labeled according to the predicted human residue number, except for the helical bundle, which is entirely poly-alanine. The model was fitted into the 43S⋅IRES EM map (Hashem et al., 2013b), using automated fitting with manual adjustments in Chimera (Pettersen et al., 2004).

**Nomenclature and Residue Numbering**

Throughout the paper, we use *S. cer.* residue numbering for all data. All crosslink data are therefore mapped onto the *S. cer.* 40S and eIF3 sequences based on multiple sequence alignments. *S. cer.* ribosomal proteins are identified by their historical names as well as the newly proposed naming system (Ban et al., 2014).

**Chemical Crosslinking Coupled to Mass Spectrometry**

EIF3 samples from *L. klu.* or *L. klu.* and *D. han.* were assembled from purified, recombinant subunits as described above. 60 μl of purified 40S⋅eIF1⋅eIF3 sample was cross-linked with 1 mM disuccinimidyl suberate d0/d12 (DSS, Creative Molecules Inc.) at room temperature (RT) for 30 min and quenched for 10 min at RT with ammonium bicarbonate (50 mM final concentration). Samples assembled in high salt, where no significant ribosome oligomerization occurred, were processed immediately, while low salt samples, which showed a significant level of ribosome oligomerization, were applied to 10%–47% sucrose gradients to separate monomeric 40S fractions from oligomeric assemblies and unbound eIF3 before further processing. There are strength and weaknesses in each of these approaches, as the extra purification steps lower the final peptide yields, while the nonpurified samples retain a background of eIF3 interlinks from the unbound eIF3 fraction of the sample. To ensure a large and unbiased set of identified crosslinked peptides, we included crosslinks from both data types in the final data set, although we did exclude eIF3⋅eIF3 interlinks from our nonpurified samples, as there is no unbiased way to determine whether the crosslink was generated when eIF3 was bound to 40S or free in solution.

After 1 hr treatment with 10 μg/ml RNase A (Roche), the samples were reduced with 2.5 mM TCEP in 8 M urea at 37°C for 30 min and alkylated with 5 mM iodoacetamide (Sigma-Aldrich) for 30 min at RT in the dark. Before digestion, the samples were diluted with ammonium bicarbonate to a 1 M final concentration of urea. 2% w/w trypsin (Promega) was added overnight at 37°C and inactivated by acidification with 2% (w/v) trifluoroacetic acid (TFA). Peptides were purified with Sep-Pak C18 MicroSpin columns (Waters, Milford, MA), according to the manufacturer’s protocol, followed by enrichment of crosslinked peptides using size exclusion chromatography (Leitner et al., 2012). LC-MS/MS analysis was carried out on an Orbitrap Elite mass spectrometer (Thermo Electron, San Jose, CA) and samples were shot in duplicates. Data were searched using xQuest (Rinner et al., 2008) in iontag mode against a database containing the protein sequences of all eIF3 subunits, eIF1 and all 40S ribosomal proteins with a precursor mass tolerance of 10 ppm. For matching of fragment ions tolerances of 0.2 Da for common-ions and 0.3 Da for crosslink ions were used and only peptides with a minimal length of 4 amino acids were considered. False discovery rates (FDR) of crosslinked peptides were assigned using xProphet (Walzthoeni et al., 2012). Crosslinked peptides were identified with a delta score of < 0.95 and a linear discriminant (ld) score of > 28 as described in the text. Mass spectra of the most abundant peaks were additionally analyzed by visual inspection in order to ensure good matches of ion series for both cross-linked peptides.

**Determination of the Molecular Architecture of the 40S⋅eIF1⋅eIF3 Complex by Integrative Structural Modeling**

The integrative approach to determine the *S. cerevisiae* 40S⋅eIF1⋅eIF3 complex architecture proceeds through four stages (Alber et al., 2007a, Alber et al., 2007b, Fernandez-Martinez et al., 2012, Lasker et al., 2010a, Lasker et al., 2010b): (1) gathering of data, (2) representation of subunits and translation of the crosslinking data and the prior knowledge into a Bayesian scoring function, (3) configurational sampling to produce an ensemble of models that minimize the Bayesian scoring function, and (4) analysis of the ensemble (Figure S6). All residue ranges described below refer to the *S. cerevisiae* sequence.

**Gathering of Data**

We used the crystallographic structures of the 40S ribosome subunit (PDB 3U5F and 3U5G) as well as the eIF3 subunit domains described in the main text (the eIF3a/eIF3c PCI heterodimer, the eIF3b β propeller, and the eIF3b-CTD/eIF3i/eIF3g-NTD complex). We removed chain h (the Stm1 protein) from the 40S structure, and rigid-body adjusted the position of helix ES6A to better fit the 43S EM density (Hashem et al., 2013a). We increased the structural coverage of the complex by adding comparative models for the eIF3b-RRM (residues 37-626, human homolog PDB 2KRB) in complex with a short eIF3j fragment (residues 30-41), eIF3g-RRM (residues 183-282, human homolog, PDB 2CQ0), eIF3j-CTD helical domain (residues 146-210, human homolog, PDB 3BPJ), and eIF1 (human homolog, PDB 2IF1 (Fletcher et al., 1999)). Comparative models were built with MODELER 9.12 (Sali and Blundell, 1993). The *S. cerevisiae* eIF1⋅40S interface was modeled using the *Tetrahymena thermophila* 40S⋅eIF1 complex structure (Rabl et al., 2011). Sequence segments without a template structure included eIF3a-CTD (residues 496-964), eIF3c-NTD (residues 1-251), eIF3g linker (residues 97-183), eIF3j disordered regions (41-145) as well as other missing loops or terminal domains (Figure S7). The 965 crosslink observations (Table S4) were identified by mass spectrometry from eight independent experiments. We used the crosslink Id-Score (Rinner et al., 2008) and FDR (Walzthoeni et al., 2012) to define three crosslink confidence classes, allowing us to construct a noise model for the crosslinking data (Figures 5D, S5 and Table S5).

**Representation of Subunits**

The domains of the 40S⋅eIF1⋅eIF3 subunits were represented by beads, arranged into either a rigid body or a flexible string, based on the available crystallographic structures and comparative models (Figure S7). To balance the thoroughness of configurational sampling and precision of model representation, we represented the structures in a multi-scale fashion. For the crosslink and excluded volume scoring terms, the crystallographic structures and comparative models were coarse-grained by representing each consecutive segments of 1 and 10 residues by a bead, centered on the center of mass, respectively (Figure S7). Sequence segments missing in the crystal structures were substituted by a single or multiple beads of the corresponding size (Figure S7). For each domain and interface with an atomic model, the beads representing a structured region were kept rigid with respect to each other during configurational sampling (i.e., rigid bodies). The rigid bodies include: the 40S⋅eIF1 complex, the eIF3a/eIF3c PCI heterodimer, eIF3b(655-699)/eIF3i/eIF3g(43-86) complex, eIF3b-RRM/eIF3j(30-41), eIF3g-RRM, eIF3b short helix (631-655), eIF3g(8-38) N-terminal double-stranded β sheet, eIF3g(86-97) short helix, and the C-terminal helices eIF3j(146-167), eIF3j(168-187), and eIF3j(187-210). Segments without a crystallographic structure or comparative model (i.e., segments predicted to be disordered or structured without a known homolog) were represented by a flexible string of large beads corresponding to a maximum of 30 residues each (Figure S7).

**Bayesian Scoring Function**

The Bayesian approach (Rieping et al., 2005) estimates the probability of a model, given information available about the system, including both prior knowledge and newly acquired experimental data. The model *M* ≡ (*X*, {*α*_*i*_}) includes the structure coordinates *X* and additional parameters {*α*_*i*_} (below). Using Bayes’ theorem, the posterior probability *p*(*M*|*D,I*), given data *D* and prior knowledge *I*, is

p(M|D,I)∝p(D|M,I)p(M|I)

where the likelihood function *p*(*D*|*M,I*) is the probability of observing data *D*, given *I* and *M*; and the prior is the probability of model *M*, given *I*. To define the likelihood function, one needs a forward model that predicts the data point (i.e., the presence of a crosslink between two given residues) given the structure, and a noise model that specifies the distribution of the deviation between the observed and predicted data points. The Bayesian scoring function is the negative logarithm of *p*(*D*|*M,I*)*p*(*M*|*I*), which ranks the models identically to the posterior probability. All information gathered in Stage 1 is encoded into the Bayesian scoring function as a likelihood function (crosslinking data), as well as priors (sequence connectivity restraints to enforce proximity between beads representing consecutive sequence, and the excluded volume between all pairs of beads).

**Forward Model for Chemical Crosslinking Data**

For a given experimentally identified crosslink *d*_*n*_ between two lysines *i* and *j*, the forward model predicts the presence of the crosslink given the structural coordinates *X*. Because of the multi-scale representation, residues are mapped into coarse-grained beads with variable radius, which represents the uncertainty of their positions. Furthermore, the rigid-body representation of crystallographic structures does not account for the flexibility of residues placed in loops. Therefore, we introduced the parameters {*σ*_*k*_}, one for each crosslinked lysine, that define the unknown radii of the spheres, centered on the beads representing the lysines, within which the position of the lysine Cα is uniformly distributed. Given **r**_*i*_ and **r**_*j*_ (the coordinates of the Cα of the two lysines) and the maximum length for the crosslinker *l*_*XL*_, including the lysine side chains, we defined the forward model *f*_*n*_ as the probability of randomly picking two points ${\tilde{r}}_{i}$ and ${\tilde{r}}_{j}$ within the spheres centered on **r**_*i*_ and **r**_*j*_, with radii *σ*_*i*_ and *σ*_*j*_, such that the distance between them ${\tilde{r}}_{\mathrm{ij}}$ is lower than *l*_*XL*_. The forward model value is one (ie, the identified crosslink is present in the given structure) when the spheres are closer than *l*_*XL*_ and the radii are sufficiently small. It has a value of zero (i.e., the identified crosslink is not present in the given structure) if the distance between the spheres exceeds *l*_*XL*_. To reduce the number of parameters in the model, we utilized a single structural uncertainty *σ*, whose value estimates the average of {*σ*_*k*_} for all crosslinked residues. We estimated *l*_*XL*_ = 21 Å from potential of mean force calculations of a system comprising of the DSS crosslinker covalently attached to the two lysine sidechains (Yannick Spill and Michael Nilges, personal communication).

**Likelihood Function for the Chemical Crosslinking Data**

The likelihood function for an identified crosslink *d*_*n*_ is defined by the following data noise model:

$$p\left(d_{n}|X,I\right)=\psi_{n}\left(1-f_{n}\right)+f_{n}\left(1-\psi_{n}\right)$$

where *ψ*_*n*_ ∈[0,0.5] is the uncertainty associated with the crosslink identification. When the value of *ψ*_*n*_ is 0.5, the uncertainty is maximal and the likelihood does not depend on the forward model *f*_*n*_ or on the distance between the lysines. When *ψ*_*n*_ is 0.0, the uncertainty is minimal, and the likelihood is identical to the forward model, i.e., it reaches a maximum when the distance between the spheres *r*_*ij*_ is below the crosslink length *l*_*XL*_. The joint likelihood function *p*(*D*|*M,I*) for a data set *D* = {*d*_*n*_} of *N*_*XL*_ of independently observed crosslinks is the product of likelihood functions for each data point. Crosslinks observed multiple times are considered as independent observations, and therefore considered multiple times in the joint likelihood function. Analysis of the intra-40S crosslink distances (Figure 5C) allowed us to group the crosslinks into three classes, including a class with an estimated ∼5% false-positive rate (high confidence crosslinks with Id-Score > 36.0; FDR < 0.05), a class with a ∼10% false-positive rate (medium confidence with 32.0 < Id Score < 36.0; 0.05 < FDR < 0.1) and a class with ∼30% false-positive rate (low confidence with 28.0 < Id Score < 32.0; 0.1 < FDR < 0.3) (Figures 5D, S5 and Table S5). We reduced the number of *ψ*_*n*_ parameters, by defining uncertainties *ψ*_*H*_ (358 crosslinks), *ψ*_*M*_ (263 crosslinks) and *ψ*_*L*_ (344 crosslinks) for the three classes.

**Prior**

The model prior *p*(*M|I*) is defined as a product of the individual priors *p*(*X*), *p*(*σ*), *p*(*ψ*_*H*_), *p*(*ψ*_*M*_), and *p*(*ψ*_*L*_) on the structural coordinates, structural uncertainties, and the three data point uncertainties, respectively. The prior *p*(*X*) included the excluded volume and terms to maintain sequence connectivity. The excluded volume restraint was implemented as a pairwise hard-sphere repulsive potential, where the bead radii were determined using the statistical relationship between the volume and the number of residues (Alber et al., 2007a, Shen and Sali, 2006). The sequence connectivity terms are upper harmonic distance restraints, with a threshold distance set to be four times the sum of the radii of the two connected beads. To facilitate configurational sampling, we applied a weak restraint whose score depended linearly on the distance between cross-linked residues, with a slope of 0.01 Å^−1^. *p*(*σ*), *p*(*ψ*_*H*_), *p*(*ψ*_*M*_), and *p*(*ψ*_*L*_) are uniform distributions, that bound *σ* to interval [0,100], and the *ψ* parameters to the interval [0,5].

**Sampling Model Configurations**

Structural models of the 40S⋅eIF1⋅eIF3 complex were computed by Replica Exchange Gibbs sampling, based on Metropolis Monte Carlo sampling (Rieping et al., 2005). This sampling was used to generate a collection of configurations for the system as well as values for the uncertainty parameters. The Monte Carlo movements included random translation and rotation of rigid bodies (1 Å and 0.025 radian maximum, respectively), random translation of individual beads in the flexible segments (1 Å maximum), as well as Gaussian perturbation of the uncertainty parameters. The sampling was run on 64 replicas, with temperatures ranging between 1.0 and 2.5. Each replica produced between 10,000 and 30,000 models. 12 independent sampling calculations were run, each one starting with a random initial configuration, for a total of 180,000 models. This set of models was divided into two ensembles of the same size, to assess sampling convergence (Figure S8). Furthermore, 6 additional runs were carried to assess the robustness of the modeling approach by removing approximately 10% and 20% of the crosslinks, respectively.

**Analysis of the Model Ensemble**

For each ensemble, the solutions (ie, the 500 best scoring models) were grouped using k-means clustering using the root-mean-square deviation (rmsd) of all eIF3 subunits as a structural similarity criterion when the models were superposed on the 40S subunit structure. The rmsd distance matrix identified two dominant clusters of similar structures (Figure S8A), here named cluster 1 and 2, with cluster 1 being more populated and scoring better than cluster 2 (Figure S8). The overall precision (calculated as the average pairwise rmsd) for cluster 1 and 2 was ∼36 and ∼41 Å, respectively, for both ensembles. The superposed structures in the cluster were converted into the probability of any volume element being occupied by a given protein (that is, the ‘localization density’) (Alber et al., 2007a, Fernandez-Martinez et al., 2012) (Figure 6C). While the eIF3b/eIF3a/eIF3c/eIF3j subunits localize on the 40S surface similarly in the two clusters, the eIF3i subunit displayed two alternate positions (Figure S8B and S8C): one toward the 40S head (cluster 1) and one toward the bottom of the body (cluster 2). Localization precision calculated for each individual domain (Table S6) indicated that the eIF3b β propeller was the domain with the most precisely determined position (∼15 Å), followed by the eIF3a/c PCI and the eIF3b-RRM. eIF3j and the eIF3b helical linker positions were determined less precisely, and eIF3i localized with different precision within the two clusters (∼38 Å in cluster 1 and ∼58 Å in cluster 2). eIF3g-RRM was the least well determined component, with a localization precision of ∼90 Å.

## Author Contributions

J.P.E., F.S., R.P., and N.B. conceived the study and experimental approach; J.P.E., S.Z., and T.S. cloned, purified, and crystallized eIF3 samples, collected and processed X-ray diffraction data, solved the structures, and built the atomic models; J.P.E., T.S., F.S., and C.H.S.A. prepared samples for EM and CX-MS experiments. C.H.S.A. and D.B. collected EM data and calculated the EM reconstructions; F.S. performed CX-MS experiments; R.P. carried out the integrative modeling with the help of P.C.; J.P.E., F.S., and R.P. analyzed the integrative modeling results, and J.P.E., F.S., R.P., A.S., R.A., and N.B. wrote the paper with input from all authors.

## Accession Numbers

The Protein Data Bank accession numbers for the structures reported in this paper are 4U1D (eIF3a), 4U1C (eIF3a/eIF3c complex), 4U1F (eIF3b β propeller) and 4U1E (eIF3b-CTD/eIF3i/eIF3g-NTD complex), 3J7J (PCI⋅MPN core model docked into 43S⋅IRES EM map) and 3J7K (PCI⋅MPN core model docked into 43S EM map). The EMDB accession numbers for the EM density maps reported in this paper are 2670 (eIF3-occupied 40S) and 2671 (unoccupied 40S).

## Supplemental Information

Document S1. Tables S1–S3, S5, and S6

Table S4. Crosslinks Used in this Study, Related to Figure 5 “Id” denotes the exact amino acid sequence of the crosslinked peptide and the exact position of the crosslink, e.g., AYAVGDVEKAALVK-FNISSHPQSPKR-a9-b11 indicates that the lysine on position 9 in the first peptide was crosslinked with the lysine on position 11 in the second peptide. This is also defined as a “unique crosslink.” It is important to note, that multiple identifications of unique crosslinks within one sample, including technical replicates, were never counted, only multiple identifications of unique crosslinks in different and independent samples. “Protein1” and “Protein2” are the protein identifiers for the cross linked peptides. “Residue 1” and “Residue 2” define the position of the crosslinked lysines within the sequence of the protein. “*S. cer.*_Corr_1” and “*S. cer.*_Corr_2” define the position of the crosslinked lysines within the sequence when the protein is aligned to *S. cerevisiae*. “Frequency” defines how often a crosslinked residue pair was identified (i.e., its redundancy within our dataset). In the dataset, a unique crosslink is not simply defined by the joined lysine pair, but by the matching identity of the two linked peptides. Therefore, we can have a duplicated or redundant crosslink within the same experiment if the peptide context of a linked lysine pair is different, or alternatively, we can have redundancy between experiments if exactly matching peptides (i.e., unique crosslinks) are independently detected multiple times. “Organism” stands for the species origin of the respective sample. “Id_Score” is a weighted sum of four subscores: xcorrc, xcorrx, match-odds and TIC that is used to assess the quality and match of the composite experimental MS2 spectra with the theoretical spectra as calculated by XQuest. “Confidence Class” indicates the confidence class (high/middle/low) of a crosslink. “FDR” denotes the false-discovery rate as calculated by xProphet. “Median Distance” denotes calculated average Cα-Cα distances in angstrom between linked lysine residues for the final set of solutions from our integrative modeling approach. “Xlink_type” indicates the nature of the respective crosslink (inter- or intralink).

Document S2. Article plus Supplemental Information
