# Molecular Architecture of the 40S⋅eIF1⋅eIF3 Translation Initiation Complex

**DOI:** 10.1016/j.cell.2014.11.001

**Published:** 2014-11-20

**Authors:** Jan P. Erzberger, Florian Stengel, Riccardo Pellarin, Suyang Zhang, Tanja Schaefer, Christopher H.S. Aylett, Peter Cimermančič, Daniel Boehringer, Andrej Sali, Ruedi Aebersold, Nenad Ban

(Cell *158*, 1123–1135; August 28, 2014)

In preparing the article above, we inadvertently assigned the coordinates for eIF3f to its homolog eIF3h and vice versa within the mammalian eIF3 model during PDB database deposition. This error led to improper positioning of these subunits in Figures 3E, 7F, and S2B. We present below revised panels for Figures 3 and 7 with the correct assignment for eIF3f and eIF3h. These figures, along with Figure S2, have been corrected online. The deposited PDB files have also been corrected and updated. In addition, we have added the missing legend for Figure S2B online. We apologize for any confusion that these errors may have caused.

**Figure 3:**
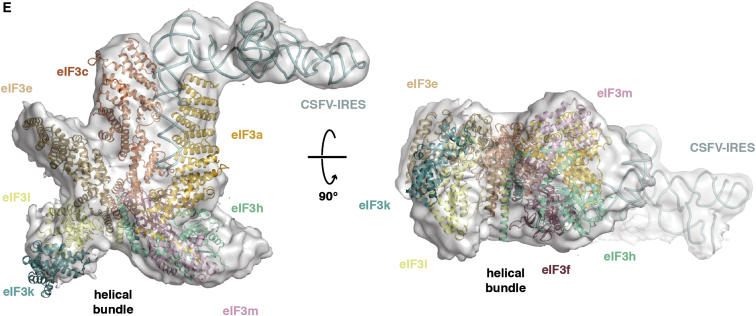
Docking of eIF3a/eIF3c in the PCI•MPN Core Density of Mammalian 43S and 43S•IRES EM Maps

**Figure 7:**
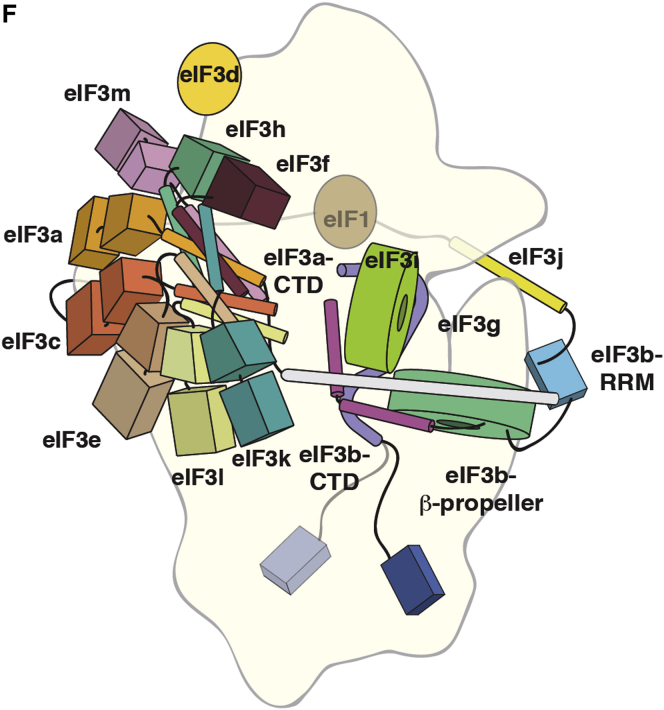
Placement and Interactions of eIF3 Components on 40S

