# Supplementary material for: Molecular Architecture of the 40S⋅eIF1⋅eIF3 Translation Initiation Complex
Source: Cell. 2014 Aug 28;158(5):1123–35. doi: 10.1016/j.cell.2014.07.044 (PMC4151992; doi:10.1016/j.cell.2014.07.044)
Supplement: Document S1. Tables S1–S3, S5, and S6 [file mmc1.pdf]

Table S1. Constructs used in this study, Related to Figure 1

Plasmid identifier	Organism	Protein	amino acid start	amino acid end
pJPE 650	*S.cerevisiae*	eIF3a / Tif32	1	494
pJPE 1003	*S.cerevisiae*	eIF3a / Tif32	228	494
pJPE 444	*S.cerevisiae*	eIF3c / Nip1	1	870
pJPE 950	*S.cerevisiae*	eIF3b / Prt1	132	626
pJPE 1060	*S.cerevisiae*	eIF3b / Prt1	655	698
pJPE 371	*S.cerevisiae*	eIF3i / Tif34	1	347
pJPE 744	*S.cerevisiae*	eIF3g / Tif35	1	135
pJPE 823	*L.kluyveri*	eIF3a / Tif32	1	854
pJPE 471	*L.kluyveri*	eIF3b / Prt1	1	736
pJPE 999	*L.kluyveri*	eIF3c / Nip1	1	808
pJPE 826	*L.kluyveri*	eIF3i / Tif34	1	349
pJPE 755	*L.kluyveri*	eIF3g / Tif35	1	281
pJPE 1104	*L.kluyveri*	eIF3j / Hcr1	1	269
pJPE 1001	*L.kluyveri*	eIF1 / Sui1	1	108
pJPE 569 (polycistronic)	*D.hansenii*	eIF3b-eIF3i-eIF3g / Prt1-Tif34-Tif35	2 (eIF3b)<br>1 (eIF3i)<br>1 (eIF3g)	736 (eIF3b)<br>349 (eIF3i)<br>276 (eIF3g)

Table S2. Data collection, phasing and refinement statistics for eIF3a and eIF3a/eIF3c, Related to Figure 2

Crystal form	Native eIF3a	Se-Met eIF3a	eIF3a/eIF3c	Se-Met eIF3a/eIF3c
Space group	C2	C2	$P3_221$	$P3_221$
Unit cell dimensions (Å)	a=242.5 b=158.3 c=93.7	a=240.8 b=156.1 c=93.3	a=b=155.55 c=91.2	a=b=155.02 c=91.2
	α =γ=90, β=104.2	α=γ=90, β=104.2	α=β=90 γ=120.00	α=β=90 γ=120
Molecules / ASU	3 monomers	3 monomers	1 heterodimer	1 heterodimer
Solvent content (%)[a]	75	75	67	67
**Data collection**				
Wavelength (Å)	1.00	0.9793	1.00	0.9793
Resolution (Å)[b]	40.0 - 3.3 (3.5 - 3.3)	40.0 - 4.0 (4.23 - 4.0)	50 – 3.5 (3.61 – 3.5)	50.0 – 3.7 (3.92-3.7)
Unique reflections[c]	48944 (7656)	55248 (8760)	16368 (2583)	26186 (4181)
Redundancy	3.3	19.4	17.8	18.8
R_{merge} (%)[d]	7.7 (82.6)	15.8 (144.2)	4.4 (162.2)	7.9 (233.6)
CC (1/2) (%)	99.8 (68.9)	99.9 (76.8)	100 (74.7)	100 (54.7)
Completeness (%)	94.2 (92.6)	98.6 (96.4)	99.9 (99.8)	99.9 (100)
I/σ	11.14 (1.53)	14.61 (2.09)	27 (2.3)	18.12 (1.59)
**Phasing**				
Se sites		22		12
FOM		0.33		0.32
**Model statistics**				
Refinement:				
resolution (Å)	40 - 3.3		45 - 3.5	
total reflections	48847		15649	
test reflections (%)	2446 (5)		1562 (10)	
R_{work} / R_{free} (%)[e]	17.03 / 20.37		26.78 / 29.79	
average B factor (Å^2)	118.1		174.8	
Rms deviations:				
bonds (Å)	0.005		0.005	
angles (°)	0.911		0.945	
Ramachandran plot:				
favored (%)	98.28		94.1	
allowed (%)	1.72		5.9	
disallowed (%)	0		0	
Molprobity clash score	13.52		9.89	
Rotamer outliers (%)	none		2.12	

[a] Estimated solvent content used during averaging/refinement.
[b] Values for highest resolution shells are given in parentheses.
[c] For natively scaled data, Bijvoet pairs were merged.
[d] $R_{merge} = \sum |I(h,i) - <I(h)>| / \sum I(h,i)$, where <I(h)> is the mean intensity of the reflections.
[e] R_{work} and R_{free} were calculated from the working and test reflection sets.

Table S3. Data collection, phasing and refinement statistics for the eIF3b-propeller and the eIF3b-CTD/eIF3i/eIF3g-NTD complex, Related to Figure 4

Crystal form	Native eIF3b	Se-Met eIF3b	eIF3b-CTD/eIF3i/eIF3g-NTD
Space group	$P2_1$	$P2_1$	$C222_1$
Unit cell dimensions (Å)	a=60.16 b=175.87 c=71.32	a=60.02 b=176.16 c=70.88	a=54.27 b=130.29 c=192.92
	α =γ=90, β=113.619	α=γ=90, β=113.366	α=β=γ=90
Molecules / ASU	2 monomers	2 monomers	1 heterotrimer
Solvent content (%)[a]	60.30	60.30	57.15
**Data collection**			
Wavelength (Å)	1.00	0.97931	1.00
Resolution (Å) [b]	50.0 - 2.2 (2.32 - 2.20)	50.0 - 2.5 (2.64 - 2.50)	50.0 – 2.0 (2.11 – 2.00)
Unique reflections [c]	67154 (10548)	89249 (14182)	46648 (7377)
Redundancy	3.1	9.7	10.4
R_{merge} (%)[d]	3.4 (64.2)	8.8 (95.1)	8.8 (199.4)
Cross correlation (%)	99.9 (71.0)	99.9 (74.6)	100 (76.4)
Completeness (%)	97.6 (97.2)	96.6 (96.4)	99.8 (99.8)
I/σ	18.51 (1.83)	18.48 (2.41)	21.25 (1.94)
**Phasing**			
Se sites		22	
FOM		0.3	
**Model statistics**			
Refinement:			
resolution (Å)	43.637 - 2.20		48.94 - 2.00
total reflections	67134		46623
test reflections (%)	4040 (6.02)		2397 (5.14)
R_{work} / R_{free} (%)[e]	18.71 / 22.62		18.58 / 21.62
average B value (Å^2)	55.30		54.11
Rms deviations:			
bonds (Å)	0.007		0.006
angles (°)	1.011		0.954
Ramachandran plot:			
favored (%)	95.5		96.5
allowed (%)	4.5		3.5
disallowed (%)	0		0
Molprobity clash score	2.05		1.76
Rotamer outliers (%)	0.7		0

[a] Estimated solvent content used during averaging/refinement.
[b] Values for highest resolution shells are given in parentheses.
[c] For natively scaled data, Bijvoet pairs were merged.
[d] $R_{merge} = \sum|I(h,i) - \langle I(h)\rangle| / \sum I(h,i)$, where $\langle I(h)\rangle$ is the mean intensity of the reflections.
[e] R_{work} and R_{free} were calculated from the working and test reflection sets.

Table S5. Statistics of mapped and calculated distances in the 40S and eIF3•40S cross-linking dataset, Related to Figure 5 and Figure 6

Statistical assessment of all unique cross-links of 40S•eIF1•eIF3 complexes analyzed by CX-MS in this study. Cα-Cα distances between linked lysine residues < 35 Å are considered "satisfied" and distances > 35 Å "violated". (A) Distribution of satisfied and validated cross-links mapped onto the X-ray structure of the yeast 40S particle grouped by Id-Score for the complete 40S dataset. (B) The same dataset as (A) grouped by FDR. (C) Distribution of interlinks between eIF3 and the 40S•eIF1 particle and links between and within different eIF3 subunits. Distances are averages of all solutions from our integrated modeling.

40S cross-links (Id-Score)

Interlinks	Satisfied	Violated	Satisfied (%)
High	170	8	96
Medium	101	29	78
Low	97	56	63
Total	368	93	80

Intralinks	Satisfied	Violated	Satisfied (%)
High	110	3	97
Medium	54	3	95
Low	59	0	100
Total	223	6	97

Cross-links	Satisfied	Violated	Satisfied (%)
High	280	11	96
Medium	155	32	83
Low	156	56	74
Total	591	99	86

40S cross-links (FDR)

Interlinks	Satisfied	Violated	Satisfied (%)
FDR < 0.05	201	20	91
FDR 0.05-0.1	58	18	76
FDR 0.1-0.3	109	55	66
Total	368	93	80

Intralinks	Satisfied	Violated	Satisfied (%)
FDR < 0.05	223	6	97
FDR 0.05-0.1	0	0	
FDR 0.1-0.3	0	0	
Total	223	6	97

Cross-links	Satisfied	Violated	Satisfied (%)
FDR < 0.05	424	26	94
FDR 0.05-0.1	58	18	76
FDR 0.1-0.3	109	55	66
Total	591	93	86

eIF3 cross-links

Interlinks	Satisfied	Violated	Satisfied (%)
High	28	5	85
Medium	24	24	50
Low	27	72	27
Total	79	101	44

Intralinks	Satisfied	Violated	Satisfied (%)
High	34	0	100
Medium	28	0	100
Low	33	0	100
Total	95	0	100

Cross-links	Satisfied	Violated	Satisfied (%)
High	62	5	93
Medium	52	24	68
Low	60	72	45
Total	174	101	63

High = Id-Score > 36
Medium = Id-Score of >32 and < 36
Low = Id-Score of > 28 and < 32

Satisfied = Lysine Cα-Cα distance < 35 Å
Violated = Lysine Cα-Cα distance > 35 Å

Table S6. Localization precision, Related to Figure 6

Domain localization precision calculated on cluster 1 and cluster 2, for the two ensembles of solutions. For each subunit domain, the precision is calculated as the average pairwise RMSD between structures in the cluster.

	Ensemble 1 Precision (Å)		Ensemble 2 Precision (Å)	
Domain	Cluster 1	Cluster 2	Cluster 1	Cluster 2
eIF3a-PCI	26.8	28	25.3	25.3
eIF3a-CTD	56.5	64.8	58.5	61.4
eIF3c-NTD	38.6	38.3	38.3	38.6
eIF3c-PCI	24.1	24.5	23.4	23.3
eIF3b-RRM	28.3	28.8	29.3	29.3
eIF3b-β-propeller	15.4	17	14.9	15.8
eIF3b-linker	39.1	46.5	39.1	46.3
eIF3i	38.2	57.3	39	58
eIF3j	46.6	47.8	47.1	47.6
eIF3g-RRM	85.8	84.2	89	87.8
